# Na^+^/K^+^-ATPase Revisited: On Its Mechanism of Action, Role in Cancer, and Activity Modulation

**DOI:** 10.3390/molecules26071905

**Published:** 2021-03-28

**Authors:** Jiří Bejček, Vojtěch Spiwok, Eva Kmoníčková, Silvie Rimpelová

**Affiliations:** 1 Department of Biochemistry and Microbiology, University of Chemistry and Technology Prague, Technická 3, 166 28 Prague 6, Czech Republic; jiri.bejcek@vscht.cz (J.B.); vojtech.spiwok@vscht.cz (V.S.); 2 Department of Pharmacology, Second Faculty of Medicine, Charles University, Plzeňská 311, 150 00 Prague, Czech Republic; eva.kmonickova@lfmotol.cuni.cz; 3 Faculty of Medicine in Pilsen, Charles University, Alej Svobody 76, 323 00 Pilsen, Czech Republic

**Keywords:** anticancer activity, cardiac glycosides, combination therapy, digoxin, digitoxin, digitoxigenin, Na^+^/K^+^-ATPase activity modulation, natural compounds, ouabain, sodium-potassium pump inhibitors

## Abstract

Maintenance of Na^+^ and K^+^ gradients across the cell plasma membrane is an essential process for mammalian cell survival. An enzyme responsible for this process, sodium-potassium ATPase (NKA), has been currently extensively studied as a potential anticancer target, especially in lung cancer and glioblastoma. To date, many NKA inhibitors, mainly of natural origin from the family of cardiac steroids (CSs), have been reported and extensively studied. Interestingly, upon CS binding to NKA at nontoxic doses, the role of NKA as a receptor is activated and intracellular signaling is triggered, upon which cancer cell death occurs, which lies in the expression of different NKA isoforms than in healthy cells. Two major CSs, digoxin and digitoxin, originally used for the treatment of cardiac arrhythmias, are also being tested for another indication—cancer. Such drug repositioning has a big advantage in smoother approval processes. Besides this, novel CS derivatives with improved performance are being developed and evaluated in combination therapy. This article deals with the NKA structure, mechanism of action, activity modulation, and its most important inhibitors, some of which could serve not only as a powerful tool to combat cancer, but also help to decipher the so-far poorly understood NKA regulation.

## 1. Introduction

Cancer is a highly variable disease in terms of its origin and biological characteristics of the affected tissues. There is a plethora of molecular targets, whose interactions with suitable molecules suppress the growth and spreading of neoplastic tissues. Each of these molecular targets has mostly a different mechanism of action.

The main role in cancer development is played by oncogenes that, when mutated, produce the corresponding proteins to a greater extent or in a form with increased or still “on” activity. Such a condition can be treated with inhibitors of the given proteins. One of such is imatinib mesylate (Gleevec^TM^), the inhibitor of the Abelson tyrosine-protein kinase 1 (ABL1) used to treat chronic myeloid leukemia [1], inhibitors of the mammalian target of rapamycin (mTOR) [2], phosphatidylinositol 3-kinase (PI3K; [3]), or newly discovered pyrazolopyrimidine-based GTPase inhibitors of K-Ras (Kirsten sarcoma virus protein) [4].

However, there are many types of cancer and, therefore, in addition to these examples, a wide range of drugs and approaches acting by different mechanisms are used to treat cancer, discussed in detail in ref. [5,6]. These include, for example, anticancer chemotherapeutics such as commonly used cisplatin and oxaliplatin, which do not target only the tumor tissue and due to this non-selectivity cause many side effects. Recently, a big effort has been made to develop a therapy targeting specifically only tumor tissue, and thus, generating minimum side-effects. One such potential targets for cancer therapy, which has attracted large attention lately, is the sodium-potassium ATPase (NKA).

NKA is an integral membrane protein localized in the cell plasma membrane of animal cells. NKA belongs to the enzyme class of translocases and it is an essential protein for maintaining ionic and osmotic balance in a eukaryotic cell. During one cycle, NKA exports three Na^+^ and imports two K^+^ ions while hydrolyzing one molecule of adenosine triphosphate (ATP). Therefore, NKA is also the key player in the transmission of nerve impulses throughout the body of a neuron [7,8]. Moreover, NKA is also responsible for the osmoregulation of Na^+^ and K^+^ ions in the hypertonic and hypotonic environment [9,10,11]. In addition to the transport function, NKA bears also a role of a receptor coupled with the Src family of nonreceptor tyrosine kinases, forming a functional complex for signal transduction [12]. This is the case when NKA is only partially inhibited and there is no significant disruption of the homeostasis of Na^+^ and K^+^ ions. Depending on the cell type, after activation by the respective ligand, NKA stimulates the proliferation of healthy cells [13,14] or, contrarily, inhibits the proliferation of tumor cells [15,16]. Due to the selective reduction of cancer cell proliferation, NKA currently represents a hot molecular target for anticancer therapy.

## 2. Na^+^/K^+^-ATPase Structure

**Figure 1:**
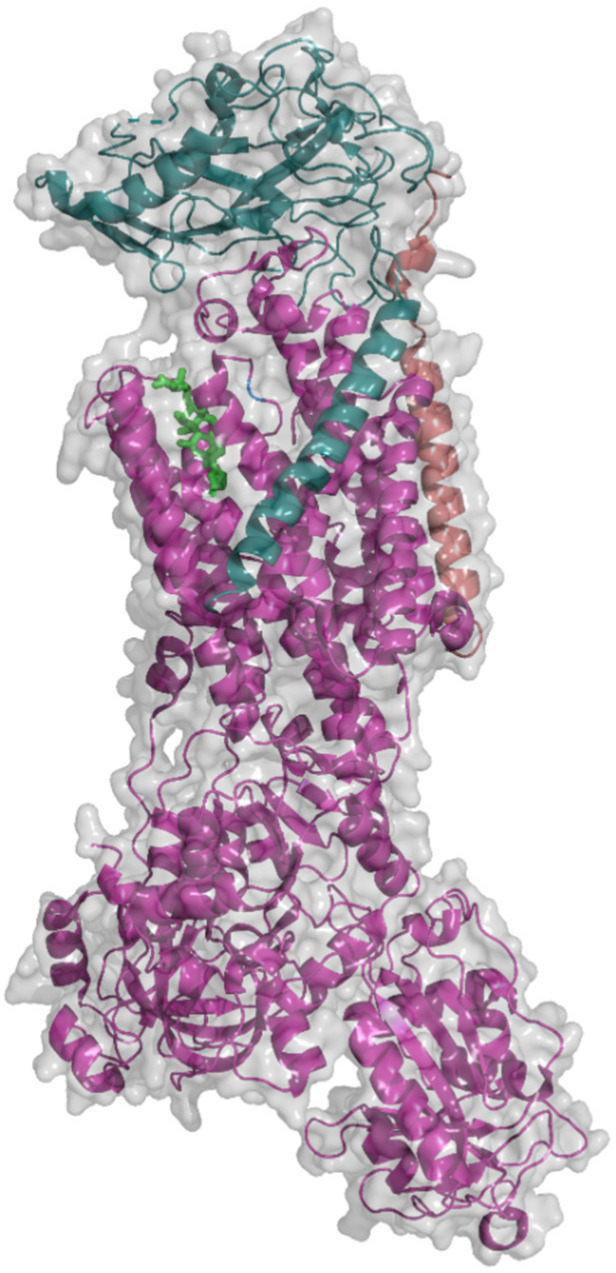
Structure of Na^+^/K^+^-ATPase (PDB, 3A3Y; [22]) with bound ouabain (green) in a molecular surface and cartoon view mode. Subunits are color-coded: Magenta (α subunit), cyan (β subunit), and orange (FXYD subunit). The image was taken using PyMOL 2.3.3.

According to the enzyme classification, NKA (Figure 1), being an integral membrane ion-transporting protein, belongs to the family of translocases (EC 7.2.2.13) utilizing the energy for ion transport from ATP hydrolysis. NKA occurs as a dimer composed of α and β subunits, which have four and three tissue-specific isoforms, respectively [17,18,19,20,21].

The α subunit is the catalytic part of the enzyme and, thus, executes all processes connected to the Na^+^ a K^+^ ion transport across the cell plasma membrane. It consists of a transmembrane and a cytoplasmic part, the second of which can be further divided into an actuator (A), nucleotide-binding (N), and phosphorylation domain (P). The spatial arrangement of the transmembrane domain (M) consists of 10 α-helices and is highly homologous with the three-dimensional structure of the transmembrane domain of another ATPase playing an important role in cancer research, the sarco-/endoplasmic reticular Ca^2+^-ATPase (SERCA) [23]. In both structures, there are the same amino acid residues at the same positions in space except for l-Asp_804_ and l-Gln_923_, which are substituted with l-Asn_804_ and l-Glu_923_ in the SERCA protein [24].

The β subunit affects the affinity of Na^+^ and K^+^ ions to their binding sites [25] and augments the level of translation of the α subunit [26]. The β subunit consists of an extracellular domain and one transmembrane helix, which interacts with transmembrane helices M_7_ and M_10_ of the α subunit. The extracellular domain of the β subunit contains three glycosylation sites [27,28], in which the asparagine residues are glycosylated by oligosaccharides containing *N*-acetylglucosamine, mannose, and partially also galactose [29]. The glycosylation level of the β subunit affects its folding and subsequent translocation into the cell plasma membrane [30]. Moreover, the β subunit also protects the α subunit from degradation, since NKA is translocated to the cell plasma membrane only as an α/β heterodimeric complex [31,32]. The glycosylation level of the β subunit does not however have importance only for the aforementioned processes, but it also plays a significant role in other events such as cellular adhesion and polarization. It was reported that in polarized hepatocytes, NKA upon deglycosylation of the β subunit translocates into the apical instead of the basolateral membrane [33]. Targeting of NKA to the basolateral membrane is important because two adjacent cells are able to form tight junctions via β subunits interaction [34].

**Figure 2:**
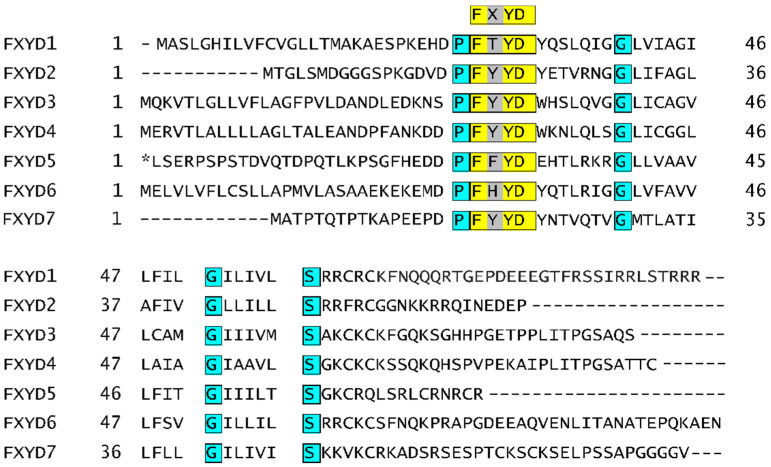
Amino acid sequence alignment of seven human isoforms of FXYD subunit of Na^+^/K^+^-ATPase. A conservative FXYD sequence is highlighted in yellow. Other shared amino acids are in turquoise. An elongated N-terminus present in isoform 5 is depicted in italics. The sequences were taken from refs. [41,42,43,44,45,46,47]. * N-terminal extension of FXYD5 isoform.

**Figure 3:**
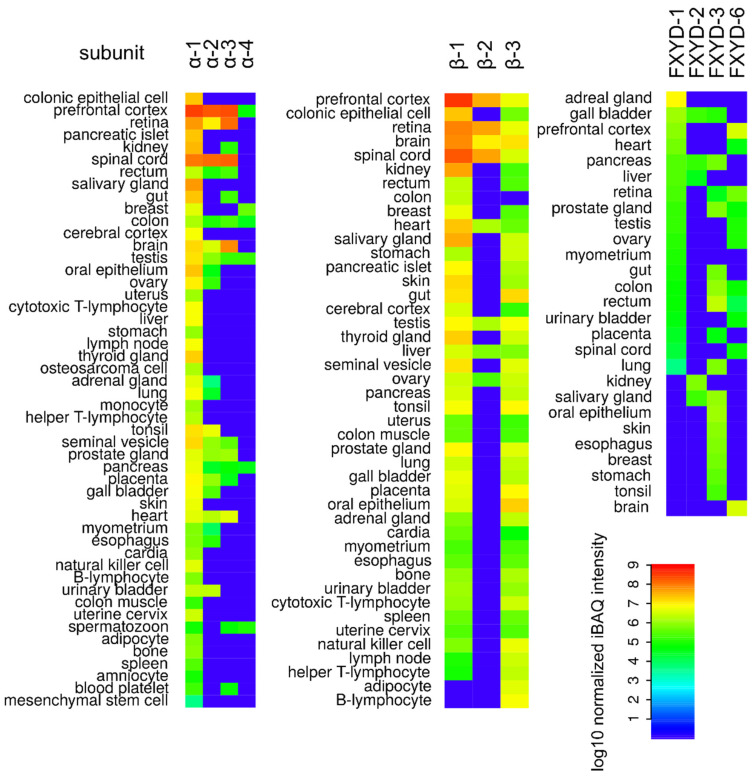
The human proteome of individual Na+/K+-ATPase isoforms of α subunit (α-1, α-2, α-3, and α-4) β subunit (β-1, β-2, and β-3) and FXYD subunit (FXYD-1, FXYD-2, FXYD-3, and FXYD-6, data for FXYD-4, FXYD-5, FXYD-7 were not available). Data were taken from ProteomicsDB [48,49,50,51,52,53,54,55,56,57,58]. The color scale represents log10 normalized intensity-based absolute quantification (iBAQ) [59] for respective isoforms in a given tissue. The plots were prepared in R software, version 3.4.4.

Even though that NKA is usually present in the form of a heterodimer of α and β subunits, at some occasions, cells produce one extra subunit designated as FXYD. The FXYD subunit comprises in total seven isoforms, which are in all cases formed by one α-helix. The FXYD subunit contains a conservative sequence, based on which this family of proteins was named. The FYD stands for L-Phe, l-Tyr, and l-Asp, respectively, X represents one of the following amino acids: l-Thr, l-Glu, l-Tyr, l-Phe, and l-His (Figure 2). Expression of the FXYD subunit is highly specific only for some tissues, mainly kidneys, heart, and muscles, summarized in Figure 3. Moreover, the expression of the FXYD subunit is also dependent on the salinity of the cell microenvironment [35]. In addition to that, a significantly increased expression of the isoform 3 of the FXYD subunit was reported for hepatocellular [36], colorectal [37], urinary bladder [38], breast carcinoma [39], and pancreatic cancer [40]. Therefore, this isoform could be conveniently utilized as a prognostic marker in the early onset of various cancer diseases.

Another role of the FXYD subunit is to stabilize NKA in the cell plasma membrane via interaction with phosphatidylserine [60]. An individual isoform of the FXYD subunit modulates the NKA activity in dependence on an affinity for Na^+^ and K^+^ ions to NKA, see summary in Table 1. Similar to the case of the β subunit, one isoform of the FXYD subunit (isoform 5) can also be glycosylated. However, contrary to the β subunit, glycosylation of the FXYD subunit reduces the level of cell adhesion by hampering transdimerization of the β subunits localized on adjacent cells [61].

## 3. Catalytic Cycle of Na^+^/K^+^-ATPase

Based on the fact that NKA similarly to SERCA transports ions against the concentration gradient, both these enzymes utilize, for the Na^+^/K^+^ and Ca^2+^, respectively, ion transport across the cell plasma membrane, energy that is supplied by a transfer of inorganic phosphate from the ATP molecule to l-Asp [70,71]. Both proteins interconvert between two conformational states called E1 and E2. The phosphorylation of l-Asp is characteristic for all types of these enzymes, based on which they previously belonged to the P-type ATPase class of enzymes (3.6.3.-).

The catalytic cycle of NKA is represented by the Post-Albers scheme, a model proposing how NKA transports the Na^+^ and K^+^ ions. During one cycle, three intracellular Na^+^ are transported into the extracellular space and two extracellular K^+^ in the opposite direction into the intracellular space via hydrolysis of one ATP molecule. The NKA catalytic cycle consists of the following steps:

- In the E_1_ state with bound ATP, the Na^+^ binding site in NKA is opened to the intracellular space and NKA has a high affinity for Na^+^ in this state.
- NKA phosphorylation occurs only when all Na^+^ binding sites are occupied since binding of the third Na^+^ ion causes a conformational change in the transmembrane domain, which is subsequently transferred to the nucleotide-binding domain [72].
- Then, after NKA phosphorylation, another conformational change takes place leading to the opening of the NKA cavity to the extracellular space, i.e., to the E_2_ state, and a release of Na^+^ [73].
- In the E_2_ state, NKA has a higher affinity for K^+^ ions. Upon their binding, NKA dephosphorylates and binds another molecule of ATP, which promotes the opening of the NKA cavity to the intracellular space, conversion to E_1_ state, and release of K^+^ ions [74,75].

By computational simulations, it was also shown that the NKA affinity to the corresponding ions is regulated in the E_1_ and E_2_ states by amino acid protonation (l-Asp_804_, l-Asp_808_, l-Asp_926_, l-Glu_327_, l-Glu_779_, l-Glu_954_) in the active site of the enzyme [76]. Moreover, it was also found that the NKA catalytic cycle is affected by Mg^2+^ ions in the way that in the E_1_ state, the Mg^2+^ ions induce the cavity occlusion, followed by autophosphorylation and transition to the E_2_ state [77]. These data are further confirmed by the computational simulations by [78], who used molecular docking and simulations of molecular dynamics to show that Mg^2+^ facilitates NKA transition from the open to occluded conformation and subsequent autophosphorylation.

There are, in total, three binding sites (I, II, III) for Na^+^ and K^+^ ions localized in the transmembrane domain of NKA between the following helices: αM_4_, αM_5_, and αM_6_ (I and II) and αM_5_, αM_6_, and αM_8_ (III). The binding sites I and II are identical for K^+^ and Na^+^. However, the binding site III is highly selective only for Na^+^ ions and occurs only in the E_1_ state [76]. As aforementioned, the ion selectivity is reached by distinct protonation states in the individual phases of the catalytic cycle [76]. This fact was confirmed by [79], who also found that the protonation of l-Asp_926_ is driven by Cl^−^ ion binding, but the exact binding site has not been uncovered yet. Besides, l-Asp_926_ is an important amino acid residue necessary for the formation of the binding site III, since its deprotonation enables Na^+^ binding, and on the contrary, its protonation causes K^+^ transition into the binding sites I and II [79].

The catalytic cycle occurs at the physiological Na^+^ and K^+^ ion concentrations; however, interestingly, it was also reported that at low Na^+^ and K^+^ concentrations, NKA can also transport H^+^ ions. In such a case, the NKA activity highly depends on the pH value, growing with decreasing pH. During this catalytic cycle, two H^+^ ions are transported to the extracellular space and then two H^+^ ions inside the cell upon hydrolysis of one ATP molecule [80].

## 4. Na^+^/K^+^-ATPase Functions and Anticancer Potential of Cardiac Steroids

**Figure 4:**
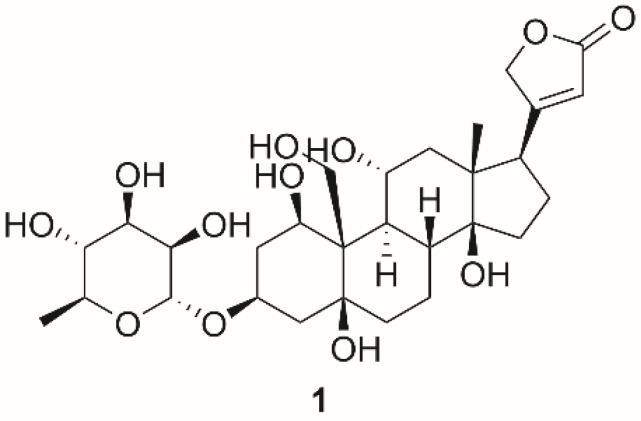
Chemical structures of ouabain (**1**).

As mentioned above, the main role of NKA is to maintain the homeostasis of Na^+^ and K^+^ ion concentrations, by which it significantly contributes to osmoregulation and maintenance of the resting membrane potential. Besides, NKA function is associated with cellular signaling resulting from its interaction with cardiac steroids (CSs). CSs are substances of a steroid character naturally occurring in sundry plants, mainly from the genus *Digitalis*, and organisms. However, there are also endogenous CSs, such as ouabain (**1**; Figure 4) and dihydroouabain [81]. The endogenous CSs are probably the reason that NKA contains in its structure the binding site for these compounds, which our body produces at picomolar to low nanomolar concentrations. At these low concentrations, NKA is not inhibited, but on the contrary, its activity is stimulated or NKA can also act as a signal transducer. Stimulation of NKA activity was observed in cardiac myocytes derived from humans, canines, and guinea pigs. The NKA activity stimulation was detected in an isoform-specific manner, which means that the most sensitive to this stimulation was α_2_ isoform [82]. Kundmiri et al. [83,84] have associated low concentrations of compound **1** with activation of NKA signaling cascades and consequently increased proliferation of opossum kidney cells as well as with stimulation of NKA ion transport. Therefore, this mechanism could be the way that the body copes with low sodium levels. Indeed, elevated amounts of compound **1** in plasma have been found in patients with low sodium [85]. The ability of compound **1** to cause organ hypertrophy and to increase cell proliferation has been reported by many authors so far, and it is probably the reason that compound **1** is often found in the plasma of patients with increased blood pressure [86,87].

It has long been known that a certain part of NKA is complexed with the non-receptor tyrosine kinase (Src kinase, SrcK), caveolin, and the epidermal growth factor receptor, which altogether form a signalosome in the caveolae of the cytoplasmic membrane. In such a case, the NKA lacks its transport function and, conversely, acquires a signaling function [88,89]. SrcK is inactive in this complex, however after the interaction of CSs with NKA, dissociation of SrcK and NKA occurs. SrcK subsequently phosphorylates the epidermal growth factor receptor [90,91] and a subsequent cascade of events results in stimulation of cell proliferation. This mechanism was demonstrated in a study in which SrcK activation occurred after stimulation of cells derived from autosomal-dominant polycystic kidney disease with ouabain (Figure 4)—a major representative of CSs broadly used in experimental pharmacology [92]. The same effect was also observed in venous endothelial cells [93]. Furthermore, in the case of reduced NKA production in prostate, breast, and kidney tumors and subsequent metastases, there was an increase in SrcK activity followed by induction of cell proliferation [94]. Thus, to induce cell proliferation by SrcK, it must not be in a complex with NKA, and this condition can be achieved by the aforementioned ways. This statement is also supported by the fact that treatment of cells with a synthetic peptide (pNaKtide) mimicking NKA reduces SrcK activity [95]. Newly, an effect of pNaKtide on the regulation of aging [96], lipid accumulation, and with it associated obesity [97], as well as suppression of steatohepatitis and atherosclerosis in mice, has also been recently discovered [98]. All these phenomena have been observed in connection with the production of reactive oxygen species (ROS), which are also associated with SrcK activation. ROS play a role in signal transduction from NKA to SrcK and, thus, it probably forms a self-amplifying loop. The importance of ROS for SrcK activation has been also demonstrated by Wang et al. [99], who reported successful SrcK activation in porcine kidney cells with hydrogen peroxide. An elevated amount of ROS causes NKA carbonylation, which possibly regulates signaling by modifying the interaction of NKA with signalosome partners [100]. Carbonylation was observed after stimulation of porcine kidney cells by compound **1**. Contrary to that, decarbonylation of the A domain of NKA occurred after the removal of compound **1** from the cell culture medium [101]. The interaction of NKA with Src kinase is mediated by two domains in both proteins. The first one is localized in the A domain of NKA and the SH2 domain of Src, and the second one is in the N domain of NKA and the kinase domain of Src [102]. The mutual interaction of NKA with Src is also likely to be dependent on the NKA isoform. It was found that isoform α-2 of NKA cannot bind to Src unless its sequence does not contain residues responsible for the interaction of Src with isoform α-1 [102].

Furthermore, in the presence of compound **1**, the NKA signalosome activates phospholipase C, which then cleaves phosphatidylinositol 4,5-bisphosphate to inositol 1,4,5-triphosphate (IP_3_). IP_3_ interacts with the IP_3_ receptor and this interaction results in oscillations of cytosolic Ca^2+^ [103,104]. These oscillations subsequently induce the synthesis of the antiapoptotic subunit p65 of nuclear factor kappa B and antiapoptotic factor B-cell lymphoma-extra-large (Bcl-xL) [105] and the activation of calmodulin-dependent kinase 2G, which inactivates the proapoptotic protein Bcl2-associated agonist of cell death (BAD) [106]. All of these effects were observed in noncancerous cells derived from renal tissue.

Another mechanism of NKA signal transduction that is independent of SrcK is the activation of PI3K, which suppresses cell motility by actin restructuralization and promotes cell adhesion with βS NKA [107,108]. However, some authors also reported that the processes associated with cell adhesion and the formation of tight and gap junctions are also linked to NKA/SrcK signaling [109], or even that this pathway promotes cell migration and healing [110]. This means that this mechanism has not been reliably elucidated yet.

**Figure 5:**
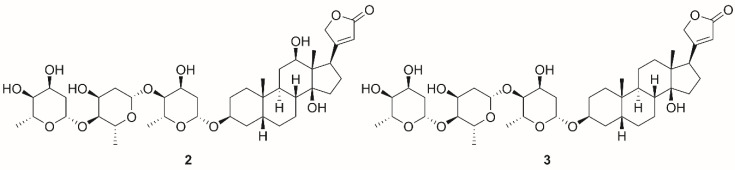
Chemical structures of digoxin (**2**) and digitoxin (**3**).

**Figure 6:**
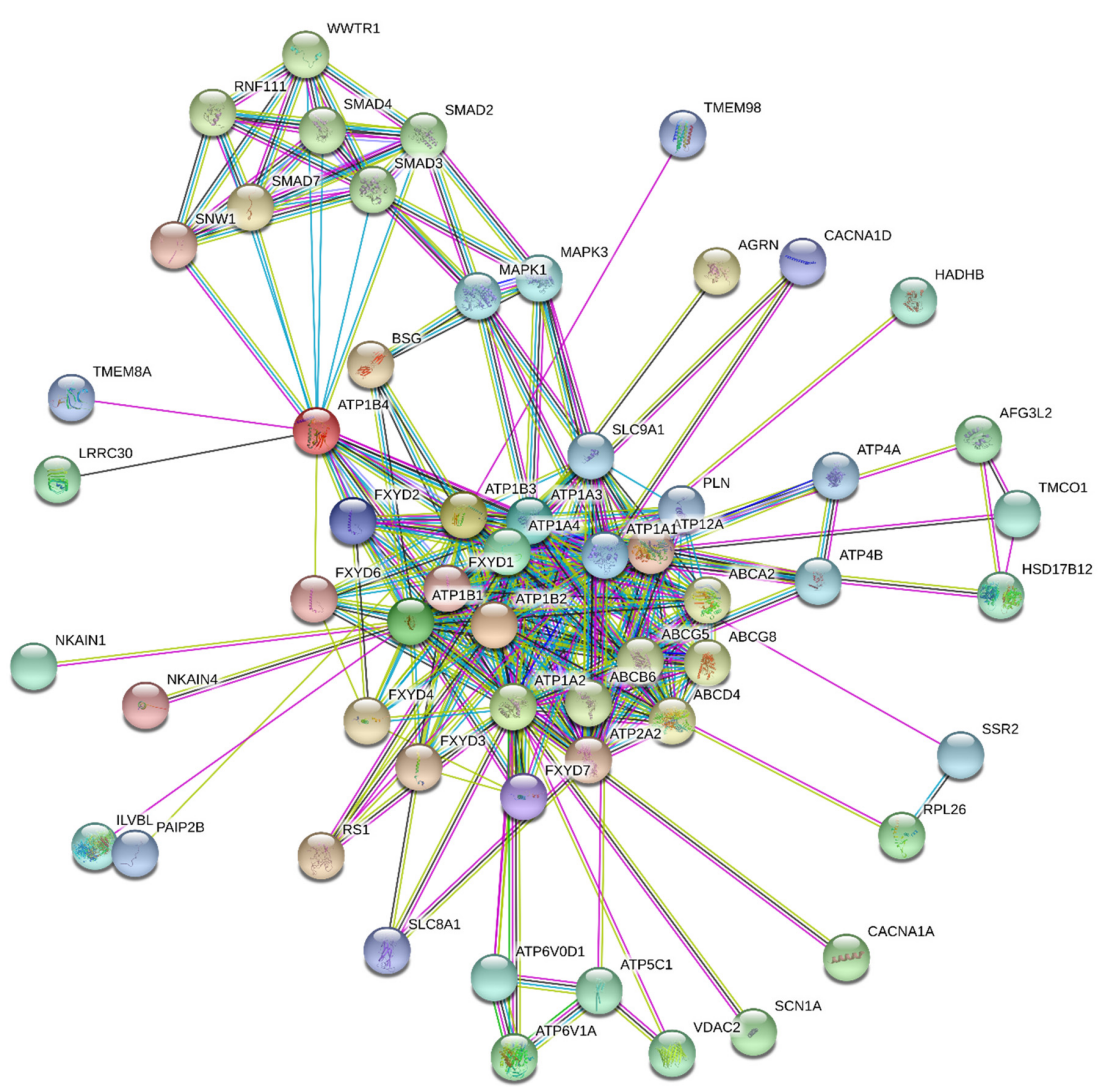
Predicted functional association network for sodium-potassium ATPase (NKA) isoforms (ATP1B4, ATP1B2, ATP1B3, ATP1A2, ATP1B1, ATP1A4, ATP1A3, ATP1A1) created by STRING 11.0 database [128]. The nodes represent gene products depicted in an evidence view mode. The type of the lines indicates knowledge or prediction of the protein-protein associations: Turquoise = from curated databases, pink = experimentally determined, green = gene neighborhood, red = gene fusions, blue = gene co-occurrence, yellow = text mining, black = co-expression, violet = protein homology. The NKA isoforms association network was generated for *Homo sapiens* species with the confidence score set to 0.700 with a maximum of 50 interactions. Small and large nodes represent proteins with unknown and known or predicted 3D structures, respectively. A description of the listed gene products is in Supplementary Information Table S1.

Another phenomenon, which has also not been reliably explained, yet is the mechanism by which NKA contributes to its anticancer activity. From the facts described above, in particular the induction of cell proliferation and antiapoptotic action, at first glance, it might seem that NKA may not be a suitable target for tumor therapy, in which the antiproliferative effects and proapoptotic action are highly desired. Originally, it was described that CSs exhibit proapoptotic effects via NKA inhibition at orders of magnitude higher concentrations, at which a positive inotropic effect in the heart occurs [111]. This results in the long-term increase in intracellular concentration of Ca^2+^, which leads to apoptosis. However, this mechanism occurring at higher CS concentrations is not selective for cancer cells and proceeds in both cancerous and noncancerous cells [112,113]. Further in-depth studies provided new data about the different effects of CSs in normal and cancer cells. Very low concentrations of CSs induce cell proliferation in noncancerous cells caused by SrcK activation but apoptosis is induced in cancer cell lines [15]. Interestingly, anticancerous effects via the activation of signal transduction occur at very low concentrations (picomolar to low nanomolar) of several tested CSs, which are below the concentrations causing NKA inhibition [15]. Kometiani et al. reported cell cycle arrest of breast cancer cells after their treatment with compound **1** (concentrations were far below the half-maximal inhibitory concentration [IC_50_]), which was caused by activation of the SrcK pathway and subsequently increased levels of the cell cycle inhibitor p21^cip1^ (cyclin-dependent kinase inhibitor 1) [114]. In a different study, digoxin (**2**, Figure 5) exhibited a cytotoxic effect in several cell lines derived from lung tumors; however, this activity was caused by SrcK and PI3K inhibition, not by SrcK activation [115]. Further, in another study, compound **1** was able to cause SrcK inhibition in human cells derived from lung adenocarcinoma (A549), which led to downregulation of focal adhesion kinase (FAK), underlining the fact that interaction of CSs with NKA in cancer cells leads to different outcomes than in non-transformed cells [116]. FAK plays role in regulating cell motility as was in the work of Pongrakhananon et al. [117], where compound **1** reduced tumor cell motility of lung cancer cells by reducing the activity of FAK, which could lead to hampering of metastatic potential of cancer cells. Reduced activity of FAK and subsequently reduced cell motility could not only reduce metastasis but could also lead to inhibition of angiogenesis as was reported by several researchers [118,119,120]. This is supported by the work of Trenti et al. [121], who reported concentration-dependent inhibition of migration of HUVEC cells after treatment with compound **1**. Angiogenesis is also closely related to hypoxia-inducible factors, a group of transcription factors that regulate the expression of angiogenic genes [122]. One of those factors is hypoxia-inducible factor 1 alpha (HIF-1α), which was shown to regulate the expression of vascular endothelial growth factor (VEGF) and angiopoietin/Tie-2 system [123]. It was discovered that protein synthesis of HIF-1α and its downstream target VEGF is inhibited by compounds **1** and digitoxin (**3**, Figure 5) leading to reduced invasive capabilities of affected cells and consequently to the slower formation of new blood vehicles [124,125,126]. Antiproliferative effects of compounds **1** and **2** used for the treatment of cells derived from colorectal carcinomas were also demonstrated in connection with NKA and volume-regulated anion channels (VRAC), since after stimulation of NKA with CSs, VRAC is opened, cell volume is reduced, the consequence of which cell proliferation is inhibited. It is also worth mentioning that these effects were not observed in a noncancerous cell line of human fibroblasts (Hs68) [127]. Therefore, it is clear that the interaction of CSs with NKA and the subsequent activation of signaling cascades has an antitumor effect, but the mechanism of action differs not only between noncancerous vs. cancerous cells but also among various cancer types. Furthermore, NKA is not only associated with the aforementioned signal cascades, but also with many other cellular processes. Some of these associated processes can be predicted by databases such as the STITCH 5.0 database, and the interactions are depicted in Figure 6. Taken together, the anticancer mechanism of CSs is dependent on their concentration. At higher concentrations (above IC_50_), they inhibit NKA by which they disrupt the ion homeostasis leading to apoptosis. On the other hand, at concentrations far below IC_50_, CSs activate several signaling pathways that are involved in their anticancer action. These actions also depend on the cancer type and include the aforementioned processes such as retention of the cell cycle by p21^cip1^, activation or inhibition of SrcK, regulation of cell volume by VRAC, and inhibition of cell motility, and angiogenesis.

## 5. Regulation of Na^+^/K^+^-ATPase Activity

### 5.1. Exogenous NKA Modulators

The most well-known NKA effectors modulating its activity are CSs, the chemical structure of which contains a steroid skeleton substituted with a lactone and saccharide moiety at the positions C-17 and C-3, respectively. As mentioned in Chapter 4, CSs can modulate NKA activity and are among the main exogenous effectors of this protein. The binding site for CSs is located in the M domain among the M_1_–M_6_ helices with the highest affinity in the P-E_2_ state, i.e., with released Na^+^ and not yet bound K^+^ [129]. The cavity, into which the steroid skeleton of CSs is bound, consists of a hydrophobic surface comprising amino acids l-Ile_315_, l-Phe_316_, l-Gly_319_ (M_4_), l-Phe_783_, l-Phe_786_ (M_5_), and l-Leu_793_ (loop M_5–6_) and hydrophilic surface composed of amino acids l-Gln_111_ (M_1_), l-Glu_117_, l-Asp_121_, l-Asn_122_ (M_2_), and l-Thr_797_ (M_6_) [130]. Of the aforementioned, amino acid residues l-Gln_111_, l-Asn_122_, and l-Thr_797_ are the most important for CS binding, as their substitution significantly reduces the sensitivity of NKA to CSs, as evidenced by numerous mutagenesis studies [131,132,133,134].

**Figure 7:**
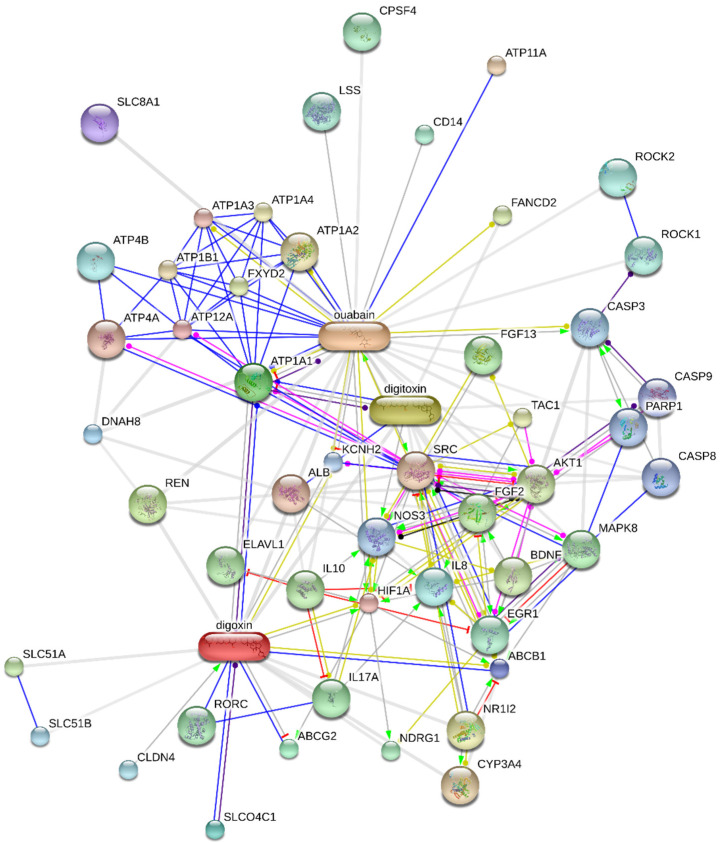
Predicted functional association network for cardiac steroids digoxin, digitoxin, and ouabain created by STITCH 5.0 database [135]. The nodes represent gene products depicted in a molecular action view. The type of the lines indicates the predicted mode of action: Green = activation, blue = binding, turquoise = phenotype, black = reaction, red = inhibition, dark blue = catalysis, pink = posttranslational modification, yellow = transcriptional regulation, a line with an arrowhead = positive, a line with a vertical bar = negative, a line with a filled circle = unspecified interaction. The cardiac steroid association network was generated according to the known and predicted interactions for *Homo sapiens* with the confidence score set to 0.700 with a maximum of 50 interactions. Small and large nodes represent proteins with unknown and known or predicted 3D structures, respectively. A description of the listed gene products is in Supplementary Information Table S2.

Dominant CS representatives are compounds **1**, **2**, and **3**. Besides NKA, these compounds can interact with a large variety of targets, some of which are depicted in Figure 7. Compounds **1**, **2**, and **3**, are currently the most widely used to study the interaction of CSs with NKA, as well as for the development of novel inhibitors. The most important element of the CS structure is the steroid core motif substituted by a lactone at C-17 and by a carbohydrate at C-3. It is exactly the structure of these three parts that are used in the development of novel NKA inhibitors or for the interaction studies.

**Figure 8:**
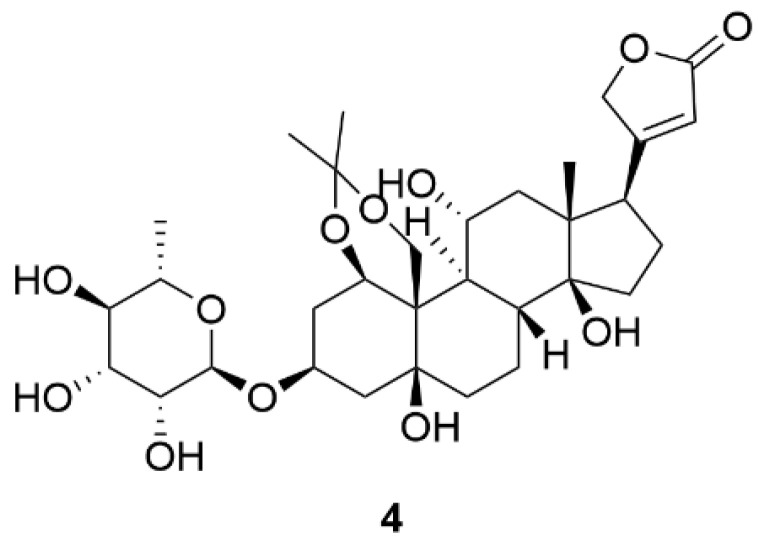
Chemical structure of compound **4**.

Appropriate distribution of hydroxyl groups on the steroid skeleton of CSs is important for their binding to NKA. The NKA binding pocket for CSs consists of a polar and non-polar part. Correspondingly, the structure of the CS steroid skeleton can be divided into polar and nonpolar surfaces. This fact is most evident in compound **1**, which, in addition to the conservative hydroxyl group at C-14, also contains hydroxyl groups at C-1, C-5, C-11, and C-19 positions and, thus, exhibits a greater in vitro NKA inhibition in comparison to compounds **2** and **3** [136]. The importance of polar interactions is evidenced by the work of Magpusao et al. [137], who blocked the hydroxyl groups of compound **1** at C-1 and C-19 positions using an acetonide group yielding a derivative **4** (Figure 8), the IC_50_ of which increased almost 120-fold in comparison to compound **1** in NKA activity assay.

Another structural motif also significantly involved in the CS binding to NKA is the lactone at the C-17 position. Its derivatization usually leads to a reduction in the NKA inhibitory effects of the resulting derivative, both in the case of double bond saturation and cycle opening [138,139] as well as by the introduction of a benzylidene group at the C-21 position [140,141]. However, in some cases, this modification resulted in a change in the CS affinity for the individual NKA isoforms [142]. This fact was also confirmed by the work of [143], who reported on specific inhibition of the α-4 isoform of NKA caused by a newly prepared derivative of compound **1**, in which the lactone group was replaced by a benzotriazole moiety, and whose IC_50_ on NKA (α-4 isoform) was three orders of magnitude lower than that for compound **1**.

Glycosylation of the hydroxyl at the C-3 position also alters the strength of CS interaction with NKA and its resulting inhibitory properties. Iyer et al. [144] investigated the effect of *O*-glycoside substitution for MeON-neoglycoside in the structure of compound **3** (Figure 5) as well as the effect of the glycosylation level of this CS on the NKA inhibitory potential. In both groups (*O*-glycosides and MeON-neoglycosides), the NKA inhibitory potency of compound **3** derivatives increases with the descending level of glycosylation. Furthermore, compared to *O*-glycosylated derivatives of compound **3**, MeON-neoglycosided compounds exhibited reduced cytotoxicity in vitro. A similar effect of CS glycosylation level on NKA inhibition was also reported by Elbaz et al. [145]. Although there is a correlation of increasing inhibitory activity of CSs towards NKA with a decreasing level of CS glycosylation, this does not apply to CS aglycones. It has been reported that an aglycone of a glycosylated form of a CS exhibits reduced NKA inhibition even when compared to triglycosylated forms of CSs [136]. The glycosylated forms of compounds **1**, **2**, and **3** also exhibit different affinities for the individual NKA isoforms, whereas the aglycones themselves do not [146]. Of the aforementioned NKA inhibitors, only compound **2** is currently under clinical evaluation as a potential drug for cancer treatment. Clinical trials including compound **2** are performed at phase I and II of clinical testing for therapy of various types of cancer (prostate, breast, pancreas, head and neck, lung, etc.) are summarized in Table 2. Not surprisingly, compound **2** is a good candidate for repurposing as a potential anticancer compound. Long-term experience of clinicians with the administration of compound **2** in cardiac diseases and collecting clinical data can overcome pharmacokinetics weaknesses of this compound, mainly the narrow therapeutic window. Moreover, results of research focusing on its antitumor effects show that low nanomolar concentrations are needed to affect molecular structures in cancer cells.

However, it seems that the biology of individual types of tumors (solid or others) offers different molecular pathways and targets for anticancer intervention of many CSs. Compounds **1**–**3**, and also other less known CSs isolated from plants, were intensively studied in the two past decades to show the anticancer mechanism in detail. Based on many individual experiments, new findings have emerged, i.e., the inhibition of NKA is transduced and continues by changes in intracellular signaling pathways. Next to Src kinase signaling described above, p38-mitogen-activated protein kinase (MAPK) cascade, PI3K/protein kinase B (Akt)/mTOR pathway, and p21^Cip1,^ or cholesterol homeostasis are also linked to α and β subunits of NKA. Specifically, for compound **2**, we can conclude that inhibition of Src signaling cascade, inhibition of hypoxia-inducible factor-1 alpha (HIF-1α) synthesis, and inhibition of androgen-dependent/independent mechanisms are the main modes of CS action for non-small-cell lung cancer, hepatoma Hep3B cells, and prostate cancer cells, respectively [147]. In clinical trials currently being performed [148], the investigation of compound **2** in the treatment of predominantly solid tumors (prostate, breast, pancreas, head and neck, lung, and melanoma) continues. Some clinical trials examine the efficacy of monotherapy of compound **2**, as has been designed for the treatment of Kaposi’s sarcoma. HIF-1α is the major regulator of tumor growth in this type of sarcoma. Other trials are related to combinatory therapy with other anticancer drugs as a currently recruiting clinical trial [149] in patients with resectable pancreatic cancer. Other studies are focused on pharmacokinetic parameters and interactions in combinatory therapy of malignant melanoma [150].

**Figure 9:**
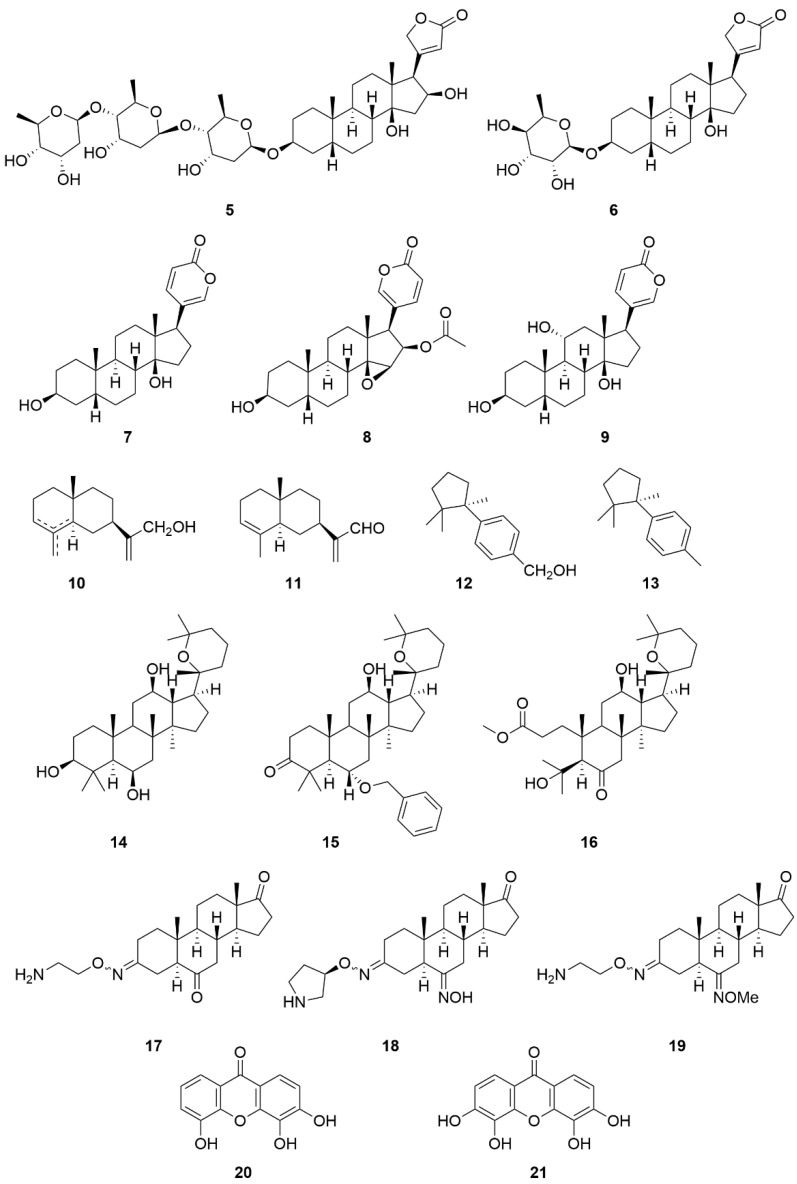
Chemical structures of selected cardiac steroids and related compounds **5**–**21**.

Apart from compound **1**, **2**, and **3**, other substances belonging to the group of CSs are also mentioned in the literature as NKA inhibitors, e.g., gitoxin (**5**), evomonoside (**6**), bufalin (**7**), cinobufagin (**8**), and gamabufotalin (**9**) (Figure 9) [136,152]. Although CSs are the best-known NKA inhibitors, other groups of compounds with the ability to inhibit this protein have been discovered lately. These compounds are characterized by condensed aromatic or saturated rings, so we may speculate similar pharmacophore as for CSs in NKA inhibition. These compounds are sesquiterpenes (**10**–**13**, Figure 9) isolated from *Thujopsis dolabrata*. Some compounds from this group exhibit anticancer and antimicrobial activity [153], and, more recently, the ability to also inhibit NKA was discovered. Their structure–activity relationship has not yet been fully elucidated. However, the hydroxymethylene group at the C-11 position of compound **10** is important for higher NKA inhibitory activity. When this group is replaced by an aldehyde group, the NKA inhibitory activity of the resulting derivative, i.e., compound **11**, decreases almost four times. Moreover, the substitution of the hydroxymethylene group (compound **12**) with a methyl group leads to loss of NKA inhibitory activity (compound **13**) [154].

Furthermore, panaxatriol (**14**, Figure 9) and its derivatives (**15**, **16**, Figure 9), belonging to the group of triterpenes, are similar to the structure of CSs since they share a steroid skeleton. The inhibitory potency of compound **14** can be further increased by the introduction of a benzyl ether moiety (compound **15**, a three-time increase) and also by opening the A ring (compound **16**, a four-time increase) [155].

Istaroxime (**17**, Figure 9) is a synthetic derivative derived from 5α,14α-androstane with the ability to inhibit NKA [156]. Istaroxime is also known for its anticancer effect [157]. Later, other derivatives based on the structure of compound **17** with enhanced NKA inhibitory activity were developed. Two of these derivatives (**18**, **19**, Figure 9) exhibited significantly higher inhibitory activity compared to compound **17**. This was achieved by replacement of linear 2-aminoethyl oxime at the C-3 position by (3*R*)-3-pyrrolidinyloxyimino chain and by introducing an oxime moiety in the C-6 position [158]. Another group of NKA inhibitors comprises substances derived from xanthone (compounds **20**, **21**, Figure 9), the potency of which to inhibit NKA depends on the number and position of hydroxyl groups; it increases with the higher hydroxyl group number. However, methylation of hydroxyl groups, on the other hand, leads to a reduction of their NKA inhibitory activity [159]. Xanthone derivatives have also shown anticancer effects [160]. However, it is difficult for these molecules as well as other compounds in Figure 9 to distinguish between the anticancer effect caused by NKA inhibition and by modulations of other targets, e.g., the nuclear receptors.

Inhibitory constants of various NKA inhibitors from Figure 9 are given in Table 3.

### 5.2. Endogenous NKA Modulators

In addition to exogenous modulators of NKA activity, there are also endogenous modulators. These compounds are present and synthesized directly in the human body and include cyclic adenosine monophosphate (cAMP). The cAMP molecule activates NKA via a signaling cascade that includes two independent pathways. The first one involves the activation of protein kinase A, while the second one activates the exchange of protein directly activated by cAMP (EPAC) 1 and 2. In both pathways, the next step is switching on the p21-activated kinase 4, which in turn triggers NKA [161]. Apart from cAMP, another endogenous NKA modulator is epinephrine (adrenaline), which induces the production of prostaglandin E2 (PGE2). Production of PGE2 ultimately leads to nitric oxide synthesis followed by the production of cyclic guanosine monophosphate and activation of protein kinase G, which results in NKA inhibition by its phosphorylation [162,163]. Besides, insulin can also regulate/activate NKA through activation of Src and extracellular signal-regulated kinases [164].

In Chapter 4, it has been shown that ROS regulates the activity of SrcK in an NKA signalosome; however, ROS also regulates the NKA pumping activity. Alharbi et al. [165] showed that ROS induced a decrease in the NKA activity, which led to apoptosis of canine cancer cells, and this effect was abrogated by pretreatment with an antioxidant N-acetylcysteine. The mechanism for this regulation of NKA activity lies in the S-glutathionylation of l-Cys residues, which leads to conformational change preventing ATP binding and thus reducing NKA activity [166,167]. However, Bibert et al. showed that the FXYD3 subunit can reverse this glutathionylation since its l-Cys residues are glutathionylated instead [168]. Another regulatory mechanism of NKA occurs during hypoxia and on the other hand, can be prevented upon reoxygenation. When cells lack oxygen, they tend to save energy by downregulating several ATP-consuming proteins, including NKA. This regulation is mediated by mitochondrial ROS and activation of protein kinase C-ζ, which leads to activation of clathrin-dependent endocytosis of NKA and degradation by the ubiquitin-conjugating system [169,170,171].

## 6. Conclusions

Cancer research has made a giant leap in recent decades owing to which, now, we not only better understand the cause of individual cancer diseases, but we also have better knowledge on systematic design of novel anticancer agents, which could have improved performance in terms of selectivity than the drugs currently used by clinicians. Recently, several research groups reported that one of cell essential enzymes, the sodium-potassium ATPase, could be a valid target in cancer treatment and that some of the natural compounds, from the group of CSs, are very potent inhibitors of NKA and when used at nontoxic low nanomolar concentrations, even selective for cancer cells. The selectivity of these compounds is probably caused by the binding of the α-subunit of NKA. However, further research needs to be done to better understand the anticancer mechanism of action of CSs. What is more, CS NKA inhibitors are very potent inducers of immunogenic cell death, by which they activate the immune system, based on which the therapy is far more efficient. Some of the CSs are used in clinical practice already from 1975 for the treatment of cardiac failure. Therefore, a possible repositioning of these compounds for other indications, such as cancer, is new hope for patients suffering from this severe disease. In addition, CSs could be used either alone or in a combination with other antineoplastic treatment methods. Thus, not only CSs, but also their molecular target, NKA, are more than valuable for further investigation. In conclusion, we hope that CSs and their semi-synthetic derivatives could be a new way for combating multidrug-resistant tumors.

## Figures and Tables

**Table 1:** The affinity of individual Na^+^/K^+^-ATPase (NKA) isoforms of FXYD subunits for Na^+^ and K^+^ ions.

Isoform	Affinity for Ions		References
	Na^+^	K^+^	
1 ^a^	+/−	n.e. ^b^	[62,63]
2	n.e.	+	[64]
3	-	-	[65]
4	+	-	[66]
5	+	-	[67]
6 ^c^	+/−	+/−	[68]
7 ^c^	+/−	n.e.	[69]

^a^ the affinity for Na^+^ depends on the level of NKA phosphorylation. ^b^ n.e.—not effective. ^c^ the affinity for Na^+^ and K^+^ depends also on the α and β isoforms.

**Table 2:** Clinical trials of digoxin (**2**) for cancer treatment; the data were taken from [151].

Clinical Trial Identifier	Study Title	Condition or Disease	First Posted	Status	Phase	Intervention/ Treatment
NCT02906800	Potentiation of cisplatin-based chemotherapy by digoxin in advanced unresectable head and neck cancer patients	Head and neck cancer	20 September 2016	Unknown	I, II	**Digoxin**
NCT04094519	A study to evaluate the effect of multiple doses of enzalutamide on the pharmacokinetics of substrates of P-glycoprotein (digoxin) and breast cancer resistant protein (rosuvastatin) in male subjects with prostate cancer	Prostate cancer	19 September 2019	Active, not recruiting	I	Enzultamide, enzultamide placebo, **digoxin**, rosuvastatin
NCT01763931	DIG-HIF-1 pharmacodynamic trial in newly diagnosed operable breast cancer	Breast cancer	9 January 2013	Completed	II	**Digoxin**
NCT01162135	Digoxin for recurrent prostate cancer	Prostate cancer	14 July 2010	Completed	II	**Digoxin**
NCT01887288	Capecitabine with digoxin for metastatic breast cancer	Metastatic breast cancer	26 June 2013	Terminated	II	Capetabine, **digoxin**
NCT00650910	Study to examine the effects of lapatinib on the pharmacokinetics of digoxin in subjects w/ErbB2 positive breast cancer	Neoplasm, breast	2 April 2008	Completed	I	Lapatinib, **digoxin**
NCT03889795	Phase IB metformin, digoxin, simvastatin in solid tumors	Advanced pancreatic cancer, advanced solid tumor	26 March 2019	Recruiting	I	Metformin, simvastatin, **digoxin**
NCT03928210	Digoxin induced dissolution of CTC clusters	Breast cancer, circulating tumor cells (CTCs)	26 April 2019	Not yet recruiting	I	**Digoxin**
NCT04141995	FOLFIRINOX with digoxin in patients with resectable pancreatic cancer	Pancreas cancer, adenocarcinoma of the pancreas	28October 2019	Not yet recruiting	II	**Digoxin**, 5-fluorouracil, calcium leucovorin, irinotecan, oxaliplatin
NCT02106845	Effect of regorafenib on digoxin and rosuvastatin in patients with advanced solid malignant tumors	Neoplasms	8 April 2014	Completed	I	**Digoxin**, rosuvastatin, regorafenib
NCT04322552	A pharmacokinetic interaction study between apatinib mesylate and transporter Pgp substrate digoxin in advanced solid tumor subjects	Advanced solid tumor	26 March 2020	Recruiting	I	Apatinib mesylate, **digoxin**
NCT01517399	Drug-drug interaction study of tivantinib (ARQ 197) with omeprazole, S-warfarin, caffeine, midazolam, and digoxin in cancer subjects	Solid tumors	25 January 2012	Completed	I	Tivantinib, omeprazole, s-warfarin, caffeine, vitamin K (dietary supplement), **digoxin**, midazolam
NCT02626234	A drug-drug interaction (DDI) study to assess the effect of INC280 on the pharmacokinetics of digoxin and rosuvastatin in patients with cMET-dysregulated advanced solid tumors	cMET-dysregulated Advanced Solid Tumors	10 December 2015	Completed	I	INC280, **digoxin**, rosuvastatin
NCT00281021	Second line erlotinib (Tarceva) plus eigoxin in non-small cell lung cancer	Carcinoma, non-small cell lung	24 January 2006	Terminated	II	Erlotinib, **digoxin**
NCT01765569	A pharmacokinetic study to investigate the effect of vemurafenib on Digoxin in Patients With BRAFV600 mutation-positive Metastatic Melanoma	Malignant melanoma, neoplasms	10 January 2013	Completed	I	**Digoxin**, vemurafenib
NCT02740712	Pharmacokinetic drug-drug interaction study of rucaparib (DDI)	Neoplasms	15 April 2016	Completed	I	Caffeine, warfarin, omeprazole, midazolam, **digoxin**, vitamin K, rucaparib
NCT02212639	Phase II multicentric study of digoxin per os in classic or endemic Kaposi’s sarcoma (KADIG 01)	Classic and endemic Kaposi’s sarcoma, lymph angio proliferations	8 August 2014	Unknown	II	Digoxin
NCT03720366	A study of perpetrator drug interactions of enasidenib in AML patients	Leukemia, myeloid, acute	25 October 2018	Not yet recruiting	I	Caffeine, dextromethorphan, flurbiprofen, midazolam, omeprazole, **digoxin**, rosuvastatin, pioglitazone
NCT03684772	Topical ionic contra-viral therapy in actinic keratosis	Actinic keratosis	26 September 2018	Recruiting	II	ICVT topical gel, furosemide topical, **digoxin** topical gel, vehicle topical gel
NCT02138292	A phase 1B clinical trial of trametinib plus digoxin in patients with unresectable or metastatic BRAF wild-type melanoma	Melanoma	14 May 2014	Completed	I	Trametinib, **digoxin**
NCT02915666	A clinical trial of patients with melanoma	Melanoma	27 September 2016	Withdrawn	I	**Digoxin** combination, dabrafenib, trametinib
NCT02732275	DS-3201b in participants with lymphomas	Lymphoma, malignant, non-Hodgkin lymphoma	8 April 2016	Recruiting	I	DS-3201b, midazolam, **digoxin**
NCT02333643	A phase 2 efficacy study of CLS003 ICVT in subjects with cutaneous warts	Cutaneous warts	7 January 2015	Completed	II	CLS003, furosemide, **digoxin**, vehicle topical

**Table 3:** Inhibitory constants (K_i_) of selected Na^+^/K^+^-ATPase inhibitors.

Compound Name (Code)	Group of Compounds	K_i_ or IC_50_ [μM]	Isoform/Source	Ref.
Ouabain (**1**)	Cardiac steroids	0.09 ± 0.01	Shark (rectal gland microsomes, α_3_)	[136]
Digoxin (**2**)		0.13 ± 0.02		
Digitoxin (**3**)		0.18 ± 0.01		
**4**		12.4 *	Porcine cerebral cortex	[137]
Gitoxin (**5**)		0.16 ± 0.04	Shark (rectal gland microsomes, α_3_)	[136]
Evomonoside (**6**)		0.11 ± 0.01		
Bufalin (**7**)		0.13 ± 0.00		
Cinobufagin (**8**)		0.68	Pig kidney	[152]
Gamabufotalin (**9**)		0.16 ± 0.02	Shark (rectal gland microsomes, α_3_)	[136]
**10**	Sesquiterpenes	55.62 ± 0.41	Porcine cerebral cortex	[154]
**11**		212.0 ± 1.92		
**12**		108.09 ± 2.01		
**13**		>494.22		
Panaxatriol (**14**)	Triterpenes	1.09 ± 0.11	Human Na^+^/K^+^-ATPase	[155]
**15**		0.33 ± 0.03		
**16**		0.26 ± 0.03		
Istaroxime (**17**)	Steroids	0.11	Dog kidney	[158]
**18**		0.02		
**19**		0.02		
3,4,5-trihydroxyxanthone (**20**)	Hydroxyxanthones	10.0	Dog kidney	[159]
3,4,5,6-tetrahydroxyxanthone (**21**)		1.5		

**4**. 4-((3*R*,3a*R*,5*R*,5a*S*,5b*R*,9a*R*,11*S*,12a*S*,14a*R*,14b*S*)-5,12a,14b-trihydroxy-3a,8,8-trimethyl-11-(((2*R*,3*R*,4*R*,5*R*,6*S*)-3,4,5-trihydroxy-6-methyltetrahydro-2*H*-pyran-2-yl)oxy)hexadecahydro-6*H*-cyclopenta[7,8]phenanthro[4,4a-*d*][1,3]dioxin-3-yl)furan-2(5*H*)-one; **10**. mixture of: a) 2-((2*R*,4a*R*,8a*R*)-4a,8-dimethyl-1,2,3,4,4a,5,6,8a-octahydronaphthalen-2-yl)prop-2-en-1-ol, b) 2-((2*R*,4a*R*,8a*S*)-4a-methyl-8-methylenedecahydronaphthalen-2-yl)prop-2-en-1-ol, c) 2-((2*R*,4a*R*)-4a,8-dimethyl-1,2,3,4,4a,5,6,7-octahydronaphthalen-2-yl)prop-2-en-1-ol; **11**. 2-((2*R*,4a*R*,8a*R*)-4a,8-dimethyl-1,2,3,4,4a,5,6,8a-octahydronaphthalen-2-yl)acrylaldehyde; **12**. (*R*)-(4-(1,2,2-trimethylcyclopentyl)phenyl)methanol; **13**. (*R*)-1-methyl-4-(1,2,2-trimethylcyclopentyl)benzene; **15.** (5*R*,6*S*,8*R*,10*R*,12*R*,13*R*,14*R*,17*S*)-6-(benzyloxy)-12-hydroxy-4,4,8,10,14-pentamethyl-17-((*R*)-2,6,6-trimethyltetrahydro-2*H*-pyran-2-yl)hexadecahydro-3*H*-cyclopenta[*a*]phenanthren-3-one; **16**. methyl 3-((3*S*,3a*R*,4*R*,6*R*,7*R*,9a*R*,9b*R*)-4-hydroxy-7-(2-hydroxypropan-2-yl)-6,9a,9b-trimethyl-8-oxo-3-((*R*)-2,6,6-trimethyltetrahydro-2*H*-pyran-2-yl)dodecahydro-1*H*-cyclopenta[a]naphthalen-6-yl)propanoate; **18**. (5*S*,6*E*,8*R*,9*S*,10*R*,13*S*,14*S*)-3-((2-aminoethoxy)imino)-6-(methoxyimino)-10,13-dimethylhexadecahydro-17*H*-cyclopenta[*a*]phenanthren-17-one; **19**. (5*S*,6*E*,8*R*,9*S*,10*R*,13*S*,14*S*)-6-(hydroxyimino)-10,13-dimethyl-3-((((*R*)-pyrrolidin-3-yl)oxy)imino)hexadecahydro-17*H*-cyclopenta[*a*]phenanthren-17-one, * *p* = 0.0002.

## Supplementary Materials

The following are available online, Table S1: A description of the listed gene products from STRING database; Table S2: A description of the listed gene products from STITCH database.

## Abbreviations

ABL1	Abelson tyrosine-protein kinase 1
Akt	Protein kinase B
ATP	Adenosine triphosphate
BAD	Bcl2 associated agonist of cell death
Bcl-XL	B-cell lymphoma-extra large
cAMP	Cyclic adenosine monophosphate
cGMP	Cyclic guanosine monophosphate
CSs	Cardiac steroids
EPAC	Exchange of protein directly activated by cAMP
FAK	Focal adhesion kinase
GTP	Guanosine triphosphate
HIF-1α	Hypoxia-inducible factor 1 alpha
IC_50_	Half maximal inhibitory concentration
IP_3_	1,4,5-Triphosphate
K-Ras	Kirsten sarcoma virus protein
MAPK	p38-mitogen-activated protein kinase
mTOR	Mammalian target of rapamycin
NKA	Sodium-potassium-ATPase
p21^cip1^	Cyclin-dependent kinase inhibitor 1
PI3K	Phosphatidylinositol 3-kinase
PGE2	Prostaglandin E2
ROS	Reactive oxygen species
SERCA	Sarco-/endoplasmic reticular Ca^2+^-ATPase
siRNA	Small interfering ribonucleic acid
SrcK	Non-receptor tyrosine kinase
VEGF	Vascular endothelial growth factor
VRAC	Volume-regulated anion channels

## References

[1] Castagnetti F., Gugliotta G., Breccia M., Stagno F., Iurlo A., Albano F., Abruzzese E., Martino B., Levato L., Intermesoli T. (2015). Long-term outcome of chronic myeloid leukemia patients treated frontline with imatinib. Leukemia.

[2] Zhang H., Berel D., Wang Y., Li P., Bhowmick N.A., Figlin R.A., Kim H.L. (2013). A comparison of Ku0063794, a dual mTORC1 and mTORC2 inhibitor, and temsirolimus in preclinical renal cell carcinoma models. PLoS ONE.

[3] FDA. https://www.fda.gov/news-events/press-announcements/fda-approves-first-pi3k-inhibitor-breast-cancer.

[4] McCarthy M.J., Pagba C.V., Prakash P., Naji A.K., van der Hoeven D., Liang H., Gupta A.K., Zhou Y., Cho K.J., Hancock J.F. (2019). Discovery of high-affinity noncovalent allosteric KRAS inhibitors that disrupt effector binding. ACS Omega.

[5] Guimarães I.S., Daltoé R.D., Herlinger A.L., Madeira K.P., Ladislau T., Valadão J.C., Lyra P.C.M., Teixeira S.F., Amorim G.M., dos Santos D.Z., Rangel L. (2013). Conventional cancer treatment. Cancer Treatment—Conventional and Innovative Approaches.

[6] Pucci C., Martinelli C., Ciofani G. (2019). Innovative approaches for cancer treatment: Current perspectives and new challenges. Ecancermedicalscience.

[7] Castillo J.P., De Giorgis D., Basilio D., Gadsby D.C., Rosenthal J.J.C., Latorre R., Holmgren M., Bezanilla F. (2011). Energy landscape of the reactions governing the Na^+^ deeply occluded state of the Na^+^/K^+^-ATPase in the giant axon of the humboldt squid. Proc. Natl. Acad. Sci. USA.

[8] Blom H., Bernhem K., Brismara H. (2016). Sodium pump organization in dendritic spines. Neurophotonics.

[9] Ferrer-Martinez A., Casado F.J., Felipe A., Pastor-Anglada M. (1996). Regulation of Na^+^,K(^+^)-ATPase and the Na^+^/K^+^/Cl^-^ co-transporter in the renal epithelial cell line NBL-1 ender osmotic stress. Biochem. J..

[10] Tang C.H., Wu W.Y., Tsai S.C., Yoshinaga T., Lee T.H. (2010). Elevated Na^+^/K^+^-ATPase responses and its potential role in triggering ion reabsorption in kidneys for homeostasis of marine euryhaline milkfish (*Chanos chanos*) when acclimated to hypotonic fresh water. J. Comp. Physiol. B.

[11] Wong M.K.S., Pipil S., Ozaki H., Suzuki Y., Iwasaki W., Takei Y. (2016). Flexible selection of diversified Na^+^/K^+^-ATPase α-subunit isoforms for osmoregulation in teleosts. Zool. Lett..

[12] Tian J., Cai T., Yuan Z., Wang H., Liu L., Haas M., Maksimova E., Huang X.Y., Xie Z.J. (2006). Binding of Src to Na^+^/K^+^-ATPase forms a functional signaling complex. Mol. Biol. Cell.

[13] Aydemir-Koksoy A., Abramowitz J., Allen J.C. (2021). Ouabain-induced signaling and vascular smooth muscle cell proliferation. J. Biol. Chem..

[14] Chen L., Jiang P., Li J., Xie Z., Xu Y., Qu W., Feng F., Liu W. (2019). Periplocin promotes wound healing through the activation of Src/ERK and PI3K/Akt pathways mediated by Na/K-ATPase. Phytomedicine.

[15] Prassas I., Karagiannis G.S., Batruch I., Dimitromanolakis A., Datti A., Diamandis E.P. (2011). Digitoxin-induced cytotoxicity in cancer cells is mediated through distinct kinase and interferon signaling networks. Mol. Cancer Ther..

[16] Khajah M.A., Mathew P.M., Luqmani Y.A. (2018). Na^+^/K^+^ ATPase activity promotes invasion of endocrine resistant breast cancer cells. PLoS ONE.

[17] Edwards I.A., Bruce G., Lawrenson C., Howe L., Clapcote S.J., Deuchars S.A., Deuchars J. (2013). Na^+^/K^+^ ATPase α1 and α3 isoforms are differentially expressed in α- and γ-motoneurons. J. Neurosci..

[18] Radzyukevich T.L., Neumann J.C., Rindler T.N., Oshiro N., Goldhamer D.J., Lingrel J.B., Heiny J.A. (2013). Tissue-specific role of the Na,K-ATPase α2 isozyme in skeletal muscle. J. Biol. Chem..

[19] Sanchez G., Nguyen A.N.T., Timmerberg B., Tash J.S., Blanco G. (2006). The Na,K-ATPase alpha4 isoform from humans has distinct enzymatic properties and is important for sperm motility. Mol. Hum. Reprod..

[20] Hundal H.S., Marette A., Ramlal T., Liu Z., Klip A. (1993). Expression of beta subunit isoforms of the Na^+^,K(^+^)-ATPase is muscle type-specific. FEBS Lett..

[21] Arystarkhova E., Sweadner K.J. (1997). Tissue-specific expression of the Na,K-ATPase beta3 subunit. The presence of beta3 in lung and liver addresses the problem of the missing subunit. J. Biol. Chem..

[22] PDB. https://www.rcsb.org/structure/3A3Y.

[23] Peterková L., Kmoníčková E., Ruml T., Rimpelová S. (2020). Sarco/endoplasmic reticulum calcium ATPase inhibitors: Beyond anticancer perspective. J. Med. Chem..

[24] Morth J.P., Pedersen B.P., Toustrup-Jensen M.S., Sørensen T.L., Petersen J., Andersen J.P., Vilsen B., Nissen P. (2007). Crystal structure of the sodium-potassium pump. Nature.

[25] Hilbers F., Kopec W., Isaksen T.J., Holm T.H., Lykke-Hartmann K., Nissen P., Khandelia H., Poulsen H. (2016). Tuning of the Na,K-ATPase by the beta subunit. Sci. Rep..

[26] Rajasekaran S.A., Gopal J., Willis D., Espineda C., Twiss J.L., Rajasekaran A.K. (2004). Na,K-ATPase β1-subunit increases the translation efficiency of the α1-Subunit in MSV-MDCK cells. Mol. Biol. Cell.

[27] Miller R.P., Farley R.A. (1988). All three potential N-glycosylation sites of the dog kidney (Na^+^ + K^+^)-ATPase beta-subunit contain oligosaccharide. Biochim. Biophys. Acta.

[28] Beggah A.T., Jaunin P., Geering K. (1997). Role of glycosylation and disulfide bond formation in the beta subunit in the folding and functional expression of Na,K-ATPase. J. Biol. Chem..

[29] Kanagawa M., Matsumoto K., Iwasaki N., Hayashi Y., Yamaguchi Y. (2013). Structural analysis of N-glycans attached to pig kidney Na^+^/K^+^-ATPase. J. Glyc. Lip..

[30] Tokhtaeva E., Munson K., Sachs G., Vagin O. (2010). N-Glycan-dependent quality control of the Na,K-ATPase β2 subunit. Biochemistry.

[31] Béguin P., Hasler U., Staub O., Geering K. (2000). Endoplasmic reticulum quality control of oligomeric membrane proteins: Topogenic determinants involved in the degradation of the unassembled Na,K-ATPase alpha subunit and in its stabilization by beta subunit assembly. Mol. Biol. Cell..

[32] Tokhtaeva E., Sachs G., Vagin O. (2009). Assembly with the Na,K-ATPase α1 subunit is required for export of β1 and β2 subunits from the endoplasmic reticulum. Biochemistry.

[33] Lian W.N., Wu T.W., Dao R.L., Chen Y.J., Lin C.H. (2006). Deglycosylation of Na^+^/K^+^-ATPase causes the basolateral protein to undergo apical targeting in polarized hepatic cells. J. Cell Sci..

[34] Vagin O., Tokhtaeva E., Sachs G. (2006). The role of the β1 subunit of the Na,K-ATPase and its glycosylation in cell-cell adhesion. J. Cell. Biol..

[35] Wang P.J., Lin C.H., Hwang H.H., Lee T.H. (2008). Branchial FXYD protein expression in response to salinity change and its interaction with Na^+^/K^+^-ATPase of the euryhaline teleost *Tetraodon nigroviridis*. J. Exp. Biol..

[36] Wang L.J., Li Q.J., Le Y., Ouyang H.Y., He M.K., Yu Z.S., Zhang Y.F., Shi M. (2018). Prognostic significance of sodium-potassium ATPase regulator, FXYD3, in human hepatocellular. Carcinoma Oncol. Lett..

[37] Widegren E., Önnesjö S., Arbman G., Kayed H., Zentgraf H., Kleeff J., Zhang H., Sun X.-F. (2009). Expression of FXYD3 protein in relation to biological and clinicopathological variables in colorectal cancers. Chemotherapy.

[38] Zhang Z., Pang S.T., Kasper K.A., Luan C., Wondergem B., Lin F., Chuang C.K., Teh B.T., Yang X.J. (2011). FXYD3: A promising biomarker for urothelial carcinoma. Biomark Insights.

[39] Herrmann P., Aronica S.M. (2015). Estrogen and tamoxifen up-regulate FXYD3 on breast cancer cells: Assessing the differential roles of ER α and ZEB1. Springerplus.

[40] Kayed H., Kleeff J., Kolb A., Ketterer K., Keleg S., Felix K., Giese T., Penzel R., Zentgraf H., Büchler M.W. (2006). FXYD3 is overexpressed in pancreatic ductal adenocarcinoma and influences pancreatic cancer cell growth. Int. J. Cancer.

[41] Uniprot. https://www.uniprot.org/uniprot/O00168.

[42] Uniprot. https://www.uniprot.org/uniprot/P54710.

[43] Uniprot. https://www.uniprot.org/uniprot/Q14802-5.

[44] Uniprot. https://www.uniprot.org/uniprot/P59646.

[45] Uniprot. https://www.uniprot.org/uniprot/Q96DB9.

[46] Uniprot. https://www.uniprot.org/uniprot/Q9H0Q3.

[47] Uniprot. https://www.uniprot.org/uniprot/P58549.

[48] Proteomics DB. https://www.proteomicsdb.org/proteomicsdb/#human/proteinDetails/P05023/expression.

[49] Proteomics DB. https://www.proteomicsdb.org/proteomicsdb/#protein/proteinDetails/56274/expression.

[50] Proteomics DB. https://www.proteomicsdb.org/proteomicsdb/#protein/proteinDetails/52946/expression.

[51] Proteomics DB. https://www.proteomicsdb.org/proteomicsdb/#protein/proteinDetails/59668/expression.

[52] Proteomics DB. https://www.proteomicsdb.org/proteomicsdb/#protein/proteinDetails/51770/expression.

[53] Proteomics DB. https://www.proteomicsdb.org/proteomicsdb/#protein/proteinDetails/53052/expression.

[54] Proteomics DB. https://www.proteomicsdb.org/proteomicsdb/#protein/proteinDetails/56697/expression.

[55] Proteomics DB. https://www.proteomicsdb.org/proteomicsdb/#protein/proteinDetails/47759/expression.

[56] Proteomics DB. https://www.proteomicsdb.org/proteomicsdb/#protein/proteinDetails/56698/expression.

[57] Proteomics DB. https://www.proteomicsdb.org/proteomicsdb/#protein/proteinDetails/60188/expression.

[58] Proteomics DB. https://www.proteomicsdb.org/proteomicsdb/#protein/proteinDetails/80311/expression.

[59] Schwanhäusser B., Busse D., Li N., Dittmar G., Schuchhardt J., Wolf J., Chen W., Selbach M. (2011). Global quantification of mammalian gene expression control. Nature.

[60] Mishra N.K., Peleg Y., Cirri E., Belogus T., Lifshitz Y., Voelker D.R., Apell H.J., Garty H., Karlish S.J.D. (2011). FXYD proteins stabilize Na,K-ATPase: Amplification of specific phosphatidylserine-protein interactions. J. Biol. Chem..

[61] Tokhtaeva E., Sun H., Deiss-Yehiely N., Wen Y., Soni P.N., Gabrielli N.M., Marcus E.A., Ridge K.M., Sachs G., Vazquez-Levin M. (2016). The *O*-glycosylated ectodomain of FXYD5 impairs adhesion by disrupting cell-cell trans-dimerization of Na,K-ATPase β1 subunits. J. Cell Sci..

[62] Cirri E., Katz A., Mishra N.K., Belogus T., Lifshitz Y., Garty H., Karlish S.J.D., Apell H.J. (2011). Phospholemman (FXYD1) raises the affinity of the human α1β1 isoform of Na,K-ATPase for Na ions. Biochemistry.

[63] Despa S., Bossuyt J., Han F., Ginsburg K.S., Jia L.G., Kutchai H., Tucker A.L., Bers D.M. (2005). Phospholemman-phosphorylation mediates the β-adrenergic effects on Na/K pump function in cardiac myocytes. Circ. Res..

[64] Silva E.C.C., Masui D.C., Furriel R.P., McNamara J.C., Barrabina H., Scofano H.M., Perales J., Teixeira-Ferreira A., Leone F.A., Fontesa C.F.L. (2012). Identification of a crab gill FXYD2 protein and regulation of crab microsomal Na,K-ATPase activity by mammalian FXYD2 peptide. Biochim. Biophys. Acta-Biomembr..

[65] Bibert S., Aebischer D., Desgranges F., Roy S., Schaer D., Kharoubi-Hess S., Horisberger J.D., Geering K. (2009). A link between FXYD3 (Mat-8)-mediated Na,K-ATPase regulation and differentiation of Caco-2 intestinal epithelial cells. Mol. Biol. Cell.

[66] Béguin P., Crambert G., Guennoun S., Garty H., Horisberger J.D. (2001). CHIF, a member of the FXYD protein family, is a regulator of Na,K-ATPase distinct from the gamma-subunit. EMBO J..

[67] Miller T.J., Davis P.B. (2008). FXYD5 modulates Na^+^ absorption and is increased in cystic fibrosis airway epithelia. Am. J. Physiol. Lung Cell Mol. Physiol..

[68] Delprat B., Schaer D., Roy S., Wang J., Puel J.L., Geering K. (2007). FXYD6 is a novel regulator of Na,K-ATPase expressed in the inner ear. J. Biol. Chem..

[69] Béguin P., Crambert G., Monnet-Tschudi F., Uldry M., Horisberger J.D., Garty H., Geering K. (2002). FXYD7 is a brain-specific regulator of Na,K-ATPase alpha 1-beta isozymes. EMBO J..

[70] Gregersen J.L., Mattle D., Fedosova N.U., Nissena P., Reinhard L. (2016). Isolation, crystallization and crystal structure determination of bovine kidney Na^+^,K^+^-ATPase. Acta Crystallogr. F Struct. Biol. Commun..

[71] Sørensen L.M.T., Møller J.V., Nissen P. (2004). Phosphoryl transfer and calcium ion occlusion in the calcium pump. Science.

[72] Schneeberger A., Apell H.J. (1999). Ion selectivity of the cytoplasmic binding sites of the Na,K-ATPase: I. sodium binding is associated with a conformational rearrangement. J. Membr. Biol..

[73] Stolz M., Lewitzki E., Bergbauer R., Mäntele W., Grell E., Barth A. (2009). Structural changes in the catalytic cycle of the Na^+^,K^+^-ATPase studied by infrared spectroscopy. Biophys. J..

[74] Castillo J.P., Rui H., Basilio D., Das A., Roux B., Latorre R., Bezanilla F., Holmgren M. (2015). Mechanism of potassium ion uptake by the Na^+^/K^+^-ATPase. Nat. Commun..

[75] Leone F.A., Furriel R.P.M., McNamara J.C., Horisberger J.D., Borin I.A. (2010). Cation transport coupled to ATP hydrolysis by the (Na, K)-ATPase: An integrated, animated model. Biochem. Mol. Biol. Educ..

[76] Rui H., Artigas P., Roux B. (2016). The selectivity of the Na^+^/K^+^-pump is controlled by binding site protonation and self-correcting occlusion. eLife.

[77] Grycova L., Sklenovsky P., Lansky Z., Janovska M., Otyepka M., Amlerd E., Teisinger J., Kubalac M. (2009). ATP and magnesium drive conformational changes of the Na^+^/K^+^-ATPase cytoplasmic headpiece. Biochim. Biophys. Acta-Biomembr..

[78] Tejral G., Sopko B., Necas A., Schoner W., Amler E. (2017). Computer modelling reveals new conformers of the ATP binding loop of Na^+^/K^+^-ATPase involved in the transphosphorylation process of the sodium pump. PeerJ.

[79] Razavi A.M., Delemotte L., Berlin J.R., Carnevale V., Voelz V.A. (2017). Molecular simulations and free-energy calculations suggest conformation-dependent anion binding to a cytoplasmic site as a mechanism for Na^+^/K^+^-ATPase ion selectivity. J. Biol. Chem..

[80] Apell H.J., Benz G., Sauerbrunn D. (2011). Proton diet for the sodium pump. Biochemistry.

[81] el-Masri M.A., Clark B.J., Qazzaz H.M., Valdes R. (2002). Human adrenal cells in culture produce both ouabain-like and dihydroouabain-like factors. Jr. Clin. Chem..

[82] Gao J., Wymore R.S., Wang Y., Gaudette G.R., Krukenkamp I.B., Cohen I.S., Mathias R.T. (2002). Isoform-specific stimulation of cardiac Na/K pumps by nanomolar concentrations of glycosides. J. Gen. Physiol..

[83] Khundmiri S.J., Amin V., Henson J., Lewis J., Ameen M., Rane M.J., Delamere N.A. (2007). Ouabain stimulates protein kinase B (Akt) phosphorylation in opossum kidney proximal tubule cells through an ERK-dependent pathway. Am. J. Physiol. Cell Physiol..

[84] Khundmiri S.J., Metzler M.A., Ameen M., Amin V., Rane M.J., Delamere N.A. (2006). Ouabain induces cell proliferation through calcium-dependent phosphorylation of Akt (protein kinase B) in opossum kidney proximal tubule cells. Am. J. Physiol. Cell Physiol..

[85] Manunta P., Messaggio E., Ballabeni C., Sciarrone M.T., Lanzani C., Ferrandi M., Hamlyn J.M., Cusi D., Galletti F., Bianchi G. (2001). Salt sensitivity study group of the Italian society of hypertension. Plasma ouabain-like factor during acute and chronic changes in sodium balance in essential hypertension. Hypertension.

[86] Manunta P., Stella P., Rivera R., Ciurlino D., Cusi D., Ferrandi M., Hamlyn J.M., Bianchi G. (1999). Left ventricular mass, stroke volume, and ouabain-like factor in essential hypertension. Hypertension.

[87] Pierdomenico S.D., Bucci A., Manunta P., Rivera R., Ferrandi M., Hamlyn J.M., Lapenna D., Cuccurullo F., Mezzetti A. (2001). Endogenous ouabain and hemodynamic and left ventricular geometric patterns in essential hypertension. Am. J. Hyper..

[88] Wang H., Haas M., Liang M., Cai T., Tian J., Li S., Xie Z. (2004). Ouabain assembles signaling cascades through the caveolar Na^+^/K^+^-ATPase. J. Biol. Chem..

[89] Liang M., Tian J., Liu L., Pierre S., Liu J., Shapiro J., Xie Z.J. (2007). Identification of a pool of non-pumping Na/K-ATPase. J. Biol. Chem..

[90] Haas M., Askari A., Xie Z. (2000). Involvement of Src and epidermal growth factor receptor in the signal-transducing function of Na^+^/K^+^-ATPase. J. Biol. Chem..

[91] Haas M., Wang H., Tian J., Xie Z. (2002). Src-mediated inter-receptor cross-talk between the Na^+^/K^+^-ATPase and the epidermal growth factor receptor relays the signal from ouabain to mitogen-activated protein kinases. J. Biol. Chem..

[92] Nguyen A.N.T., Jansson K., Sánchez G., Sharma M., Reif G.A., Blanco G. (2011). Ouabain activates the Na-K-ATPase signalosome to induce autosomal dominant polycystic kidney disease cell proliferation. Am. J. Physiol. Renal. Physiol..

[93] Tverskoi A.M., Sidorenko S.V., Klimanova E.A., Akimova O.A., Smolyaninova L.V., Lopina O.D., Orlov S.N. (2016). Effects of ouabain on proliferation of human endothelial cells correlate with Na,K-ATPase activity and intracellular ratio of Na and K. Biochemistry.

[94] Banerjee G.M., Cui X., Li Z., Yu H., Cai L., Jia X., He D., Wang C., Gao T., Xie Z. (2018). Na/K-ATPase Y260 phosphorylation–mediated Src regulation in control of aerobic glycolysis and tumor. Sci. Rep..

[95] Li Z., Zhang Z., Xie J.X., Li X., Tian J., Cai T., Cui H., Ding H., Shapiro J.I., Xie Z. (2011). Na/K-ATPase mimetic pNaKtide peptide inhibits the growth of human cancer cells. J. Biol. Chem..

[96] Sodhi K., Nichols A., Mallick A., Klug R.L., Liu J., Wang X., Srikanthan K., Goguet-Rubio P., Nawab A., Pratt R. (2018). The Na/K-ATPase oxidant amplifcation loop regulates aging. Sci. Rep..

[97] Sodhi K., Maxwell K., Yan Y., Liu J., Chaudhry M.A., Getty M., Xie Z., Abraham N.G., Shapiro J.I. (2015). pNaKtide inhibits Na/K-ATPase reactive oxygen species amplification and attenuates adipogenesis. Sci. Adv..

[98] Sodhi K., Srikanthan K., Goguet-Rubio P., Nichols A., Mallick A., Nawab A., Martin R., Shah P.T., Chaudhry M., Sigdel S. (2017). pNaKtide attenuates steatohepatitis and atherosclerosis by blocking Na/K-ATPase/ROS amplification in C57Bl6 and ApoE knockout mice fed a western diet. Sci. Rep..

[99] Wang Y., Ye Q., Liu C., Xie J.X., Yan Y., Lai F., Duan Q., Li X., Tian J., Xie Z. (2014). Involvement of Na/K-ATPase in hydrogen peroxide-induced activation of the Src/ERK pathway in LLC-PK1 cells. Free Radic. Biol. Med..

[100] Yan Y., Shapiro A.P., Mopidevi B.R., Chaudhry M.A., Maxwell K., Haller S.T., Drummond C.A., Kennedy D.J., Tian J., Malhotra D. (2016). Protein carbonylation of an amino acid residue of the Na/K-ATPase α1 subunit determines Na/K-ATPase signaling and sodium transport in renal proximal tubular cells. J. Am. Heart Assoc..

[101] Yan Y., Shapiro A.P., Haller S., Katragadda V., Liu L., Tian J., Basrur V., Malhotra D., Xie Z., Abraham N.G. (2013). Involvement of reactive oxygen species in a feed-forward mechanism of Na/K-ATPase-mediated signaling transduction. J. Biol. Chem..

[102] Yu H., Cui X., Zhang J., Xie J.X., Banerjee M., Pierre S.V., Xie Z. (2018). Heterogeneity of signal transduction by Na-K-ATPase α-isoforms: Role of Src interaction. Am. J. Physiol. Cell Physiol..

[103] Miyakawa-Naito A., Uhlén P., Lal M., Aizman O., Mikoshiba K., Brismar H., Zelenin S., Aperia A. (2003). Cell signaling microdomain with Na,K-ATPase and inositol 1,4,5-trisphosphate receptor generates calcium oscillations. J. Biol. Chem..

[104] Yuan Z., Cai T., Tian J., Ivanov A.V., Giovannucci D.R., Xie Z. (2005). Na/K-ATPase tethers phospholipase C and IP3 receptor into a calcium-regulatory complex. Mol. Biol. Cell.

[105] Burlaka I., Liu X.L., Rebetz J., Arvidsson I., Yang L., Brismar H., Karpman D., Aperia A. (2013). Ouabain protects against shiga toxin–triggered apoptosis by reversing the imbalance between Bax and Bcl-xL. J. Am. Soc. Nephrol..

[106] Panizza E., Zhang L., Fontana J.M., Hamada K., Svensson D., Akkuratov E.E., Scott L., Mikoshiba K., Brismar H., Lehtiö J. (2019). Ouabain-regulated phosphoproteome reveals molecular mechanisms for Na^+^, K^+^-ATPase control of cell adhesion, proliferation, and survival. FASEB J..

[107] Barwe S.P., Anilkumar G., Moon S.Y., Zheng Y., Whitelegge J.P., Rajasekaran S.A., Rajasekaran A.K. (2005). Novel role for Na,K-ATPase in phosphatidylinositol 3-kinase signaling and suppression of cell motility. Mol. Biol. Cell.

[108] Vilchis-Nestor C.A., Roldán M.L., Leonardi A., Navea J.G., Padilla-Benavides T., Shoshani L. (2019). Ouabain enhances cell-cell adhesion mediated by β 1 subunits of the Na^+^,K^+^-ATPase in CHO fibroblasts. Int. J. Mol. Sci..

[109] del Toro A.O., Jimenez L., Hinojosa L., Martínez-Rendón J., Castillo A., Cereijido M., Ponce A. (2019). Influence of endogenous cardiac glycosides, digoxin, and marinobufagenin in the physiology of epithelial cells. Cardiol. Res. Pract..

[110] Verdejo-Torres O., Flores-Maldonado C., Padilla-Benavides T., Campos-Blázquez J.P., Larré I., Lara-Lemus R., Perez Salazar E., Cereijido M., Contreras R.G. (2019). Ouabain accelerates collective cell migration through a cSrc and ERK1/2 sensitive metalloproteinase activity. J. Membr. Biol..

[111] Reuter H., Henderson S.A., Han T., Ross R.S., Goldhaber J.I., Philipson K.D. (2002). The Na^+^-Ca^2+^ exchanger is essential for the action of cardiac glycosides. Circ. Res..

[112] Winnicka K., Bielawski K., Bielawska A., Miltyk W. (2010). Dual effects of ouabain, digoxin and proscillaridin A on the regulation of apoptosis in human fibroblasts. Nat. Prod. Res..

[113] Pan L., Zhang Y., Zhao W., Zhou X., Wang C., Deng F. (2017). The cardiac glycoside oleandrin induces apoptosis in human colon cancer cells via the mitochondrial pathway. Cancer Chemother. Pharmacol..

[114] Kometiani P., Liu L., Askari A. (2005). Digitalis-induced signaling by Na+/K+-ATPase in human breast cancer cells. Mol. Pharmacol..

[115] Lin S.Y., Chang H.H., Lai Y.H., Lin C.H., Chen M.H., Chang G.C., Tsai M.F., Chen J.J.W. (2015). Digoxin suppresses tumor malignancy through inhibiting multiple Src-related signaling pathways in non-small cell lung cancer. PLoS ONE.

[116] Shin H.K., Ryu B.J., Choi S.W., Kim S.H., Le K. (2015). Inactivation of Src-to-ezrin pathway: A possible mechanism in the ouabain-mediated inhibition of A549 cell migration. Biomed. Res. Int..

[117] Pongrakhananon V., Chunhacha P., Chanvorachote P. (2013). Ouabain suppresses the migratory behavior of lung cancer cells. PLoS ONE.

[118] Kornberg L.J., Shaw L.C., Spoerri P.E., Caballero S., Grant M.B. (2004). Focal adhesion kinase overexpression induces enhanced pathological retinal angiogenesis. Inv. Ophthalmol. Vis. Sci..

[119] Cabrita M.A., Jones L.M., Quizi J.L., Sabourin L.A., McKay B.C., Addison C.L. (2011). Focal adhesion kinase inhibitors are potent anti-angiogenic agents. Mol. Oncol..

[120] Pedrosa A.R., Bodrug N., Gomez-Escudero J., Carter E.P., Reynolds L.E., Georgiou P.N., Fernandez I., Lees D.M., Kostourou V., Alexopoulou A.N. (2019). Tumor angiogenesis is differentially regulated by phosphorylation of endothelial cell focal adhesion kinase tyrosines-397 and -861. Cancer Res..

[121] Trenti A., Zulato E., Pasqualini L., Indraccolo S., Bolego C., Trevisi L. (2017). Therapeutic concentrations of digitoxin inhibit endothelial focal adhesion kinase and angiogenesis induced by different growth factors. Br. J. Pharmacol..

[122] Pouysségur J., Dayan F., Mazure N.M. (2006). Hypoxia signalling in cancer and approaches to enforce tumour regression. Nature.

[123] Yamakawa M., Liu L.X., Date T., Belanger A.J., Vincent K.A., Akita G.Y., Kuriyama T., Cheng S.H., Gregory R.J., Jiang C. (2003). Hypoxia-inducible factor-1 mediates activation of cultured vascular endothelial cells by inducing multiple angiogenic factors. Circ. Res..

[124] Zhang H., Qian D.Z., Tan Y.S., Lee K.A., Gao P., Ren Y.R., Rey S., Hammers H., Chang D., Pili R. (2008). Digoxin and other cardiac glycosides inhibit HIF-1α synthesis and block tumor growth. Proc. Natl. Acad. Sci. USA.

[125] Lee D.H., Oh S.C., Giles A.J., Jung J., Gilbert M.R., Park D.M. (2017). Cardiac glycosides suppress the maintenance of stemness and malignancy via inhibiting HIF-1α in human glioma stem cells. Oncotarget.

[126] Yang X.S., Xu Z.W., Yi T.L., Xu R.C., Li J., Zhang W.B., Zhang S., Sun H.T., Yu Z.Q., Xu H.X. (2018). Ouabain suppresses the growth and migration abilities of glioma U-87MG cells through inhibiting the Akt/mTOR signaling pathway and downregulating the expression of HIF-1α. Mol. Med. Rep..

[127] Fujii T., Shimizu T., Yamamoto S., Funayama K., Fujita K., Tabuchi Y., Ikari A., Takeshima H., Sakai H. (2018). Crosstalk between Na^+^,K^+^-ATPase and a volume-regulated anion channel in membrane microdomains of human cancer cells. Biochim. Biophys. Acta-Mol. Basis Dis..

[128] STRING. http://string.embl.de/.

[129] Laursen M., Gregersen J.L., Yatime L., Nissen P., Fedosova N.U. (2015). Structures and characterization of digoxin- and bufalin-bound Na^+^,K^+^-ATPase compared with the ouabain-bound complex. Proc. Natl. Acad. Sci. USA.

[130] Laursen M., Yatime L., Nissen P., Fedosova N.U. (2013). Crystal structure of the high-affinity Na^+^K^+^-ATPase-ouabain complex with Mg^2+^ bound in the cation binding site. Proc. Natl. Acad. Sci. USA.

[131] Canfield V., Emanuel J.R., Spickofsky N., Levenson R., Margolskee R. (1990). Ouabain-resistant mutants of the rat Na,K-ATPase x2 isoform identified by using an episomal expression vector. Mol. Cell. Biol..

[132] O’Brien W.J., Wallick E.T., Lingrel J.B. (1993). Amino acid residues of the Na,K-ATPase involved in ouabain sensitivity do not bind the sugar moiety of cardiac glycosides. J. Biol. Chem..

[133] Croyle M.L., Woo A.L., Lingrel J.B. (1997). Extensive random mutagenesis analysis of the Na^+^/K^+^-ATPase alpha subunit identifies known and previously unidentified amino acid residues that alter ouabain sensitivity—Implications for ouabain binding. Eur. J. Biochem..

[134] Dalla S., Swarts H.G.P., Koenderink J.B., Dobler S. (2013). Amino acid substitutions of Na,K-ATPase conferring decreased sensitivity to cardenolides in insects compared to mammals insect. Biochem. Mol. Biol..

[135] STITCH. https://stitch-db.org/.

[136] Cornelius F., Kanai R., Toyoshima C. (2013). A structural view on the functional importance of the sugar moiety and steroid hydroxyls of cardiotonic steroids in binding to Na,K-ATPase. J. Biol. Chem..

[137] Magpusao A.N., Omolloh G., Johnson J., Gascón J., Peczuh M.W., Fenteany G. (2015). Cardiac glycoside activities link Na^+^/K^+^ ATPase ion-transport to breast cancer cell migration via correlative SAR. ACS Chem. Biol..

[138] Manunta P., Hamilton B.P., Hamlyn J.M. (2001). Structure-activity relationships for the hypertensinogenic activity of ouabain: Role of the sugar and lactone ring. Hypertension.

[139] Ren Y., Ribas H.T., Heath K., Wu S., Ren J., Shriwas P., Chen X., Johnson M.E., Cheng X., Burdette J.E. (2020). Na^+^/K^+^-ATPase-targeted cytotoxicity of (+)-digoxin and several semisynthetic derivatives. J. Nat. Prod..

[140] Rocha S.C., Pessoa M.T.C., Neves L.D.R., Alves S.L.G., Silva L.M., Santos H.L., Oliveira S.M.F., Taranto A.G., Comar M., Gomes I.V. (2014). 21-Benzylidene digoxin: A proapoptotic cardenolide of cancer cells that up-regulates Na,K-ATPase and epithelial tight junctions. PLoS ONE.

[141] Alves S.L.G., Paixão N., Ferreira L.G.R., Santos F.R.S., Neves L.D.R., Oliveira G.C., Cortes V.F., Salomé K.S., Barison A., Santos F.V. (2015). 9γ-Benzylidene digoxin derivatives synthesis and molecular modeling: Evaluation of anticancer and the Na,K-ATPase activity effect. Bioorg. Med. Chem..

[142] Pessôa M.T.C., Alves S.L.G., Taranto A.G., Villar J.A.F.P., Blanco G., Barbosa L.A. (2018). Selectivity analyses of γ-benzylidene digoxin derivatives to different Na,K-ATPase α isoforms: A molecular docking approach. J. Enzyme Inhib. Med. Chem..

[143] Syeda S.S., Sánchez G., Hong K.H., Hawkinson J.E., Georg G.I., Blanco G. (2018). Design, synthesis, and *in vitro* and *in vivo* evaluation of ouabain analogues as potent and selective Na,K-ATPase α4 isoform inhibitors for male contraception. J. Med. Chem..

[144] Iyer A.K.V., Zhou M., Azad N., Elbaz H., Wang L., Rogalsky D.K., Rojanasakul Y., O’Doherty G.A., Langenhan J.M. (2010). A direct comparison of the anticancer activities of digitoxin MeON-neoglycosides and *O*-glycosides. ACS Med. Chem. Lett..

[145] Elbaz H.A., Stueckle T.A., Wang H.Y.L., O’Doherty G.A., Lowry D.T., Sargent L.M., Wang L., Dinu C.Z., Rojanasakula Y. (2012). Digitoxin and a synthetic monosaccharide analog inhibit cell viability in lung cancer cells. Toxicol. Appl. Pharmacol..

[146] Katz A., Lifshitz Y., Bab-Dinitz E., Kapri-Pardes E., Goldshleger R., Tal D.M., Karlish S.J.D. (2010). Selectivity of digitalis glycosides for isoforms of human Na,K-ATPase. J. Biol. Chem..

[147] Reddy D., Kumavath R., Barh D., Azevedo V., Ghosh P. (2020). Anticancer and antiviral properties of cardiac glycosides: A review to explore the mechanism of actions. Molecules.

[148] Clinical Trials. https://www.clinicaltrials.gov/.

[149] Clinical Trials. https://clinicaltrials.gov/ct2/show/NCT04141995.

[150] Clinical Trials. https://clinicaltrials.gov/ct2/show/NCT01765569.

[151] Clinical Trials. https://www.clinicaltrials.gov/ct2/results?recrs=&cond=cancer&term=digoxin&cntry=&state=&city=&dist=.

[152] Liang G., Chung T., Guo J., Zhang R., Xü W., Tzen J.T.C., Jiang R. (2017). Novel cinobufagin oxime ether derivatives as potential Na^+^/K^+^-ATPase inhibitors: Synthesis, biological screening and molecular docking. Chem. Res. Chin. Univ..

[153] Morita Y., Matsumura E., Tsujibo H., Yasuda M., Sakagami Y., Okabe T., Ishida N., Inamori Y. (2001). Biological activity of alpha-thujaplicin, the minor component of *Thujopsis dolabrata* SIEB. et ZUCC. var. hondai MAKINO. Biol. Pharm. Bull..

[154] Oh I., Yang W.Y., Park J., Lee S., Mar W., Oh K.B., Shin J. (2011). *In vitro* Na^+^/K^+^-ATPase inhibitory activity and antimicrobial activity of sesquiterpenes isolated from *Thujopsis dolabrata*. Arch. Pharm. Res..

[155] Wu Q., Chen P., Tu G., Li M., Pan B., Guo Y., Zhai J., Fu H. (2018). Synthesis and evaluation of panaxatriol derivatives as Na^+^, K^+^-ATPase inhibitors. Bioorg. Med. Chem. Lett..

[156] De Munari S., Cerri A., Gobbini M., Almirante N., Banfi L., Carzana G., Ferrari P., Marazzi G., Micheletti R., Schiavone A. (2003). Structure-based design and synthesis of novel potent Na^+^,K^+^-ATPase inhibitors derived from a 5α,14α-androstane scaffold as positive inotropic compounds. J. Med. Chem..

[157] Alevizopoulos K., Dimas K., Papadopoulou N., Schmidt E.M., Tsapara A., Alkahtani S., Honisch S., Prousis K.C., Alarifi S., Calogeropoulou T. (2016). Functional characterization and anti-cancer action of the clinical phase II cardiac Na^+^/K^+^ ATPase inhibitor istaroxime: In vitro and in vivo properties and cross talk with the membrane androgen receptor. Oncotarget.

[158] Gobbini M., Armaroli S., Banfi L., Benicchio A., Carzana G., Ferrari P., Giacalone G., Marazzi G., Moro B., Micheletti R. (2010). Novel analogues of Istaroxime, a potent inhibitor of Na(^+^),K(^+^)-ATPase: Synthesis, structure-activity relationship and 3D-quantitative structure-activity relationship of derivatives at position 6 on the androstane scaffold. Bioorg. Med. Chem..

[159] Zhang Z., Li Z., Tian J., Jiang W., Wang Y., Zhang X., Li Z., You Q., Shapiro J.I., Si S. (2010). Identification of hydroxyxanthones as Na/K-ATPase ligands. Mol. Pharmacol..

[160] Lin C.N., Liou S.J., Lee T.H., Chuang Y.C., Won S.J. (1996). Xanthone derivatives as potential anti-cancer drugs. J. Pharm. Pharm..

[161] Ramos-Alvarez I., Lee L., Jensen R.T. (2019). Cyclic AMP-dependent protein kinase A and EPAC mediate VIP and secretin stimulation of PAK4 and activation of Na^+^,K^+^-ATPase in pancreatic acinar cells. Am. J. Physiol. Gastrointest. Liver Physiol..

[162] Moussawi L.E., Mohamed C., Kreydiyyeh S.I. (2018). Epinephrine modulates Na^+^/K^+^ ATPase activity in Caco-2 cells via Src, p38MAPK, ERK and PGE2. PLoS ONE.

[163] Moussawi L.E., Mohamed C., Kreydiyyeh S. (2019). The epinephrine-induced PGE2 reduces Na^+^/K^+^ ATPase activity in Caco-2 cells via PKC, NF-κB and NO. PLoS ONE.

[164] Shahidullah M., Mandal A., Delamere N.A. (2017). Src family kinase links insulin signaling to short term regulation of Na,K-ATPase in nonpigmented ciliary epithelium. J. Cell. Physiol..

[165] Alharbi Y., Kapur A., Felder M., Barroilhet L., Stein T., Pattnaik B.R., Patankar M.S. (2019). Plumbagin-induced oxidative stress leads to inhibition of Na^+^/K^+^-ATPase (NKA) in canine cancer cells. Sci. Rep..

[166] Petrushanko I.Y., Yakushev S., Mitkevich V.A., Kamanina Y.V., Ziganshin R.H., Meng X., Anashkina A.A., Makhro A., Lopina O.D., Gassmann M. (2012). S-glutathionylation of the Na,K-ATPase catalytic α subunit is a determinant of the enzyme redox sensitivity. J. Biol. Chem..

[167] Juel C. (2014). Oxidative stress (glutathionylation) and Na,K-ATPase activity in rat skeletal muscle. PLoS ONE.

[168] Bibert S., Liu C.C., Figtree G.A., Garcia A., Hamilton E.J., Marassi F.M., Sweadner K.J., Cornelius F., Geering K., Rasmussen H.H. (2011). FXYD proteins reverse inhibition of the Na^+^-K^+^ pump mediated by glutathionylation of its beta1 subunit. J. Biol. Chem..

[169] Dada L.A., Chandel N.S., Ridge K.M., Pedemonte C., Bertorello A.M., Sznajder J.I. (2003). Hypoxia-induced endocytosis of Na,K-ATPase in alveolar epithelial cells is mediated by mitochondrial reactive oxygen species and PKC-zeta. J. Clin. Investig..

[170] Comellas A.P., Dada L.A., Lecuona E., Pesce L.M., Chandel N.S., Quesada N., Budinger G.R.S., Strous G.J., Ciechanover A., Sznajder J.I. (2006). Hypoxia-mediated degradation of Na,K-ATPase via mitochondrial reactive oxygen species and the ubiquitin-conjugating system. Circ. Res..

[171] Chen Z., Krmar R.T., Dada L., Efendiev R., Leibiger I.B., Pedemonte C.H., Katz A.I., Sznajder J.I., Bertorello A.M. (2006). Phosphorylation of adaptor protein-2 mu2 is essential for Na^+^,K^+^-ATPase endocytosis in response to either G protein-coupled receptor or reactive oxygen species. Am. J. Respir. Cell Mol. Biol..

